# Supplementary material for: How do time trends in inhospital mortality compare? A retrospective study of England and Scotland over 17 years using administrative data
Source: BMJ Open. 2018 Feb 21;8(2):e017195. doi: 10.1136/bmjopen-2017-017195 (PMC5855481; doi:10.1136/bmjopen-2017-017195)
Supplement: Author's manuscript [file bmjopen-2017-017195.draft_revisions.pdf]

BMJ Open

BMJ Open is committed to open peer review. As part of this commitment we make the peer review history of every article we publish publicly available.

When an article is published we post the peer reviewers' comments and the authors' responses online. We also post the versions of the paper that were used during peer review. These are the versions that the peer review comments apply to.

The versions of the paper that follow are the versions that were submitted during the peer review process. They are not the versions of record or the final published versions. They should not be cited or distributed as the published version of this manuscript.

BMJ Open is an open access journal and the full, final, typeset and author-corrected version of record of the manuscript is available on our site with no access controls, subscription charges or pay-per-view fees (http://bmjopen.bmj.com).

If you have any questions on BMJ Open's open peer review process please email
editorial.bmjopen@bmj.com

BMJ Open

In-hospital mortality is declining faster in England than in Scotland: a regression analysis of hospital admissions over 17 years

Journal:	*BMJ Open*
Manuscript ID	bmjopen-2017-017195
Article Type:	Research
Date Submitted by the Author:	07-Apr-2017
Complete List of Authors:	Aragon Aragon, Maria Jose; University of York, Centre for Health Economics<br>Chalkley, Martin; University of York, Centre for Health Economics
**Primary Subject Heading**:	Health economics
Secondary Subject Heading:	Health services research
Keywords:	HEALTH ECONOMICS, Health economics < HEALTH SERVICES ADMINISTRATION & MANAGEMENT, Quality in health care < HEALTH SERVICES ADMINISTRATION & MANAGEMENT

SCHOLARONE™
Manuscripts

For peer review only

TITLE PAGE

Title: In-hospital mortality is declining faster in England than in Scotland: a regression analysis of hospital admissions over 17 years.

Authors:

María José Aragón (MJ Aragón) – Corresponding Author
mjma504@york.ac.uk
Research Fellow
Centre for Health Economics, University of York

Martin Chalkley (M Chalkley)
martin.chalkley@york.ac.uk
Professor
Centre for Health Economics, University of York

Centre for Health Economics
Alcuin 'A' Block
University of York
Heslington, York
YO10 5DD UK

Authors' Contributions:
MJ Aragón and M Chalkley defined the research question and type of analysis required, MJ Aragón performed the statistical analysis and MJ Aragón and M Chalkley analysed the results and wrote the article.

Acknowledgements
The Hospital Episode Statistics are copyright © 1997/98 - 2013/14, re-used with the permission of The Health & Social Care Information Centre. All rights reserved.
The Scottish Morbidity Record data was used with the permission of the Information and Statistics Division Scotland (ISD).

For peer review only

Declaration of competing interests

All authors have completed the Unified Competing Interest form at www.icmje.org/coi_disclosure.pdf (available on request from the corresponding author) and Dr. Chalkley and Dr. Aragon have nothing to disclose.

Data Sharing Statement

No additional data available.

Ethics Approval

No ethics approval was required for this study.

Funding Statement

This article was developed further to the NIHR funded project HS&DR - 11/1022/19.

For peer review only

ABSTRACT

Objectives

To examine the trends in in-hospital mortality for England and Scotland over a 17-year period to determine whether and if so to what extent the time trends differ after controlling for differences in the patients treated.

Design

Analysis of retrospective administrative hospital data using descriptive aggregate statistics of trends in in-hospital mortality and estimates of a logistic regression model of individual patient-level in-hospital mortality accounting for patient characteristics, case-mix and country and year specific intercepts.

Setting

Secondary care across all hospitals in England and Scotland from 1997 to 2013.

Population

Over 190 million inpatient admissions, either electively or emergency, in England or Scotland from 1997 to 2013

Data

Hospital Episode Statistics (HES) for England and the Scottish Morbidity Record 01 (SMR) for Scotland.

Main outcome measures

Separately for two admission pathways (elective and emergency) we examine aggregate time trends of the proportion of patients who die in hospital and a binary variable indicating whether an individual patient died in hospital or survived and how that indicator is influenced by the patient's characteristics, the year and the country (England or Scotland) in which they were admitted.

Results

In-hospital mortality has declined in both countries over the period studied, for both elective and emergency admissions but has declined more in England than Scotland. The difference in trend reduction is greater for elective admissions. These differences persist after controlling for patient characteristics and case-mix.

Conclusions

Comparing data at country level suggests questions about the roles performed by or functioning of their health care systems. We found substantial differences between Scotland and England in regard to the trend reductions in in-hospital mortality. Hospital resources are therefore being deployed increasingly differently over time in these two countries for reasons that have yet to be explained.

For peer review only

STRENGTHS AND LIMITATIONS OF THIS STUDY

- The first study to use comprehensive and extensive data on hospital admissions and discharges over a long period of time to study differences in in hospital mortality.
- Establishes a different perspective on in-hospital mortality – that of variation across health care systems over time and establishes that two neighbouring countries with otherwise similar health care systems have different time paths of in-hospital mortality.
- Uses detailed administrative records to control for variation in case-mix and patient characteristics.
- It is not possible to establish the potential causes of the different trends in in-hospital reported but potential causes are established as future avenues of research.

For peer review only

INTRODUCTION

In-hospital mortality has attracted a good deal of attention and concern when used as a proxy for hospital performance (1-6). The concern stems from an inability to disentangle consequences of treatment choices from the inherently different risks that patients' medical conditions pose (7-10). This debate however distracts from the potential knowledge that can be derived by studying in-hospital mortality at a more aggregate level (11). Hospital care is costly and a key resource in addressing a population's health care needs. It has been noted that death is a "core business" of hospitals (12) and hence understanding how that core business is changing – how much of the "business" of hospitals it accounts for – is a crucial aspect of health system planning and management.

Without some reference point it is impossible to determine whether an outcome such as declining in-hospital mortality is notable, or to be expected. Comparing two otherwise similar health care systems establishes each as a reference for the other. That reference is more powerful if analysis is conducted in trends. Differences in the levels of in-hospital mortality across different jurisdictions could easily be accounted for as the consequence of unobserved differences between their populations, health care needs and service organisation. However, these unobservable factors seem likely to follow common trends, so divergence in the trends of in-hospital mortality are more challenging to explain.

This, the first study of its kind, examines the trends in in-hospital mortality for England and Scotland over a 17-year period. We establish that "death as the core business of hospitals" has been declining faster in England than in Scotland over that period.

For peer review only

METHODS

Data

In both England and Scotland data are routinely collected on hospital inpatient activity through, respectively, Hospital Episode Statistics (HES) and the Scottish Morbidity Record (SMR). Both data sources report in terms of episodes (period under the care of one consultant), which are then converted into continuous inpatient spells (CIS) corresponding to the period of care that can include transfers within and between hospitals. We construct equivalent measures of CIS for both countries and distinguish between elective (including day cases) and emergency admissions, excluding maternity and regular attenders. Both data sources report on the basis if financial years (1 April to 31 March) but for convenience we denote the financial year by its first calendar year. We examine over 190 million CIS from 1997 to 2013 using discharge information to determine whether the patient died in hospital or not.

Both data sources include the characteristics of a patient in regard to age, sex and the deprivations decile of their home address. We use these together with the Healthcare Resource Group (HRG) into which the patient's treatment fell account for both variation in case-mix.

Empirical Methods

The proportion of all CIS that end in death was calculated directly from the data sources, separately for each country, year and admission pathway.

After constructing a binary outcome variable (equal to 1 if the patient died in hospital and 0 otherwise) logistic regression analysis, separately for each admission pathway, was used to determine whether differences across jurisdictions persist after including covariates; the covariates included in the analysis were age (using five-year age bands indicators), sex (as an indicator equal to one for females), HRG indicators (there are more than 1000 different HRGs in the data) and deprivation decile indicators (1 being the most deprived and 10 the least deprived). Differences between countries were captured by country specific dummy variables and interactions between those and year dummy variables.

We ran logit regressions using Stata13.

For peer review only

RESULTS

Figure 1 shows the trend in the in-hospital mortality rate for England and Scotland for elective and emergency admissions.

[FIGURE 1 HERE]

Figure 1. In Hospital Mortality Rate. Elective (LHS) and Emergency (RHS)

Note: the y-axis scales are different; LHS is 1/10 of RHS.

In Figure 1 it is apparent that in-hospital mortality has decreased in both countries, but has done so more quickly in England than in Scotland in both emergency and elective care. Over the same period the trends of overall mortality and life expectancy have been similar in both countries (13-15), see Figure 2. Whilst overall spending per head on hospital care is higher in Scotland it has followed a similar (increasing) trend as in England (16).

[FIGURE 2 HERE]

Figure 2. Crude Mortality Rate (LHS) and Life Expectancy (RHS)

Next we describe the relative, England/Scotland, in-hospital mortality rate, again separately for Elective and Emergency admissions. Figure 3 shows the ratio of the in-hospital mortality rates for both countries, normalising to 100 the initial year, and clearly shows the relative change in the in-hospital mortality rates.

[FIGURE 3 HERE]

Figure 3. In Hospital Mortality Rate. England/Scotland with 1997/98 = 100.

Next we determine whether these crude, unadjusted differences persist once we account for the different characteristics of the patients that are being treated in the two countries, specifically; age, sex, disease proxies and deprivation level.

Table 1 shows the descriptive statistics of the variables used for the regression analysis. Over the period of analysis in-hospital mortality has been higher for emergency than for elective admissions, that emergency admissions' patients are more likely to be males, are younger, and more likely to come from the highest deprivation decile than elective admissions.

[TABLE 1 HERE]

Table 2 shows regression results, presented as relative odd ratios between England and Scotland, this presentation was chosen to simplify the results table and focus on the question of interest: is the reduction in in-hospital mortality rates different between the countries after controlling for patient and CIS characteristics?

[TABLE 2 HERE]

The results in Table 2 confirm what is observed in Figures 1 and 3, the reduction in in-hospital mortality in England has been faster than that of Scotland throughout the period, even after controlling for patient and CIS characteristics. The results are reported as odd-ratios, showing the relative difference between the two countries in each period, e.g. the first row says that in the initial year of the analysis Elective admissions were 11% lower in England than in Scotland and Emergency admissions were 3% higher. For *Electives* England starts with a lower in-hospital mortality rate

For peer review only

(coefficient in first row is less than one and significant), then there is no clear trend in the difference between the countries until there is no significant difference between the two countries (non-significant coefficient in 2001/02) and then the difference with Scotland increases over time (coefficient becomes smaller over time) until in the last year in-hospital mortality for Elective admissions in England is around one third of that in Scotland. For *Emergencies* England started with a higher in-hospital mortality rate (coefficient in top row is greater than one and significant) and the difference between the two countries first increased (coefficients become greater) and then decreased until there is no difference between them (non-significant coefficient in 2007/08) and then England's in-hospital mortality rate continues to reduce relative to that of Scotland (coefficients smaller than one and significant from 2008/09 onwards) until being around 27% lower in the last year.

For peer review only

DISCUSSION

This study shows that in-hospital mortality for both elective and emergency admissions has been on a 17-year declining trend in both England and Scotland but that trend reduction has been greater in England. This remains the case after controlling for case mix and population characteristics.

We have used comprehensive and extensive data on hospital admissions and discharges over a long period of time, providing details of more than 190 million admissions. These data have been adjusted so as to be able to compare two similar health care systems so that each can act as a benchmark for the other. Whilst we can establish the differences between the experiences of these two systems with our data, we have not established causal mechanisms for these differences.

Numerous previous studies have examined the variation of in-hospital mortality across different hospitals, focusing on the details and limitations of risk-adjustment. This study provides a different perspective – that of variation across health care systems. Whilst we cannot hope to replicate the detail or depth of previous studies that focus on particular treatments we do provide a much broader and comprehensive view.

That view suggests a number of important and unanswered questions that have great potential importance for policymakers. Why has the divergence in trend reduction in in-hospital mortality developed? In what ways are these two health care systems developing different roles for their hospitals? Should there be a concern in Scotland that in-hospital mortality is decreasing less slowly and is not substantially higher than in its near neighbour England?

Ours study cannot answer these questions – that is for future research – but we can give some insight and some clues as to the possible answers. One key difference in the development of hospital-based health care in Scotland and England over the period studied has been the reform of financing undertaken in England. This has been shown to have resulted in an expansion of activity on a per-capita basis (refs). That suggests that part of the explanation for what we have observed is that hospitals in England are treating more “less-sick” patients which would result in a lower propensity for patients to die in hospital simply by increasing the denominator. However, that seems unlikely to be the whole explanation because we have established that the reductions in mortality exist for *both* elective and emergency admissions and whereas the former would appear susceptible to “denominator” effect it is less easy to account for emergency admissions in this way. Since we adjusted for the kinds of treatments that are carried out in England and Scotland it is also difficult to account for the differences in trends in terms of changing case-mix unless our adjustment is substantially flawed because there are large unobserved differences. Basing an analysis on trends mitigates this risk because for it to affect our results requires that the unobserved differences in case mix between the two systems are changing over time. This then suggests that there are two avenues to explore further. The first is to determine whether the alternatives to care in hospital setting have diverged in the two countries. If for example alternative settings to which terminally ill patients can be discharged have expanded faster in England than in Scotland we would observe the kind of differential trend of in-hospital mortality established by our analysis. The second, more worrying possibility is that there remains some element of the different in trend that relates to the efficacy of hospital treatments in the two countries.

For peer review only

REFERENCES

1. Coombes R. Experts disagree about usefulness of hospital mortality data. BMJ : British Medical Journal. 2014;349.

2. Holland W, McKee M. Can hospital mortality comparisons ever be fit for purpose? BMJ : British Medical Journal. 2013;347.

3. Lilford R, Pronovost P. Using hospital mortality rates to judge hospital performance: a bad idea that just won't go away. BMJ. 2010;340.

4. Hogan H, Zipfel R, Neuburger J, Hutchings A, Darzi A, Black N. Avoidability of hospital deaths and association with hospital-wide mortality ratios: retrospective case record review and regression analysis. BMJ : British Medical Journal. 2015;351.

5. Black N. Assessing the quality of hospitals. BMJ. 2010;340.

6. van Gestel YR, Lemmens VE, Lingsma HF, de Hingh IH, Rutten HJ, Coebergh JW. The Hospital Standardized Mortality Ratio Fallacy: A Narrative Review. Medical Care. 2012;50(8):662–7.

7. Girling AJ, Hofer TP, Wu J, Chilton PJ, Nicholl JP, Mohammed MA, et al. Case-mix adjusted hospital mortality is a poor proxy for preventable mortality: a modelling study. BMJ Quality & Safety. 2012;21:1052-6.

8. Stewart K, Choudry MI, Buckingham R. Learning from hospital mortality. Clinical Medicine. 2016;16(6):530-4.

9. Hawkes N. Patient coding and the ratings game. BMJ. 2010;340.

10. Bottle A, Jarman B, Aylin P. Hospital Standardized Mortality Ratios: Sensitivity Analyses on the Impact of Coding. Health Services Research. 2011;46(6pt1):1741-61.

11. Farrar S, Yi D, Sutton M, Chalkley M, Sussex J, Scott A. Has payment by results affected the way that English hospitals provide care? Difference-in-differences analysis. BMJ. 2009;339.

12. Bradford E. Death is 'core business' of Scottish hospitals, study finds. BBC News19 March 2014.

13. Office for National Statistics. Vital Statistics: Population and Health Reference Tables.

14. Office for National Statistics. Life Expectancy at Birth and at Age 65 by Local Areas in England and Wales.

15. Office for National Statistics. Life Expectancy at Birth and at Age 65 for the UK and Local Areas in Scotland.

16. Hawe E, Cockcroft L. OHE Guide to UK Health and Health Care Statistics. 2013.

For peer review only

Table 1. Descriptive Statistics

	Mean	Std. Dev.	Mean	Std. Dev.
Elective	**England**		**Scotland**	
**% Died in Hospital**	0.21	4.62	0.40	6.32
**% Males**	46.86	49.90	45.31	49.78
**Age**	54.43	21.26	53.73	21.03
**% Decile 1**	10.26	30.35	12.06	32.57
**% Decile 10**	9.00	28.62	8.21	27.46
**Number of Observations**	100,945,785		9,886,856	
Emergency	**England**		**Scotland**	
**% Died in Hospital**	4.98	21.75	5.01	21.82
**% Males**	48.03	50.00	49.14	49.99
**Age**	50.55	28.32	52.60	26.39
**% Decile 1**	14.70	35.41	15.85	36.52
**% Decile 10**	7.11	25.69	6.21	24.13
**Number of Observations**	74,048,633		8,259,572	

For peer review only

Table 2. Logit Regression Results. Dependent Variable: Prob(Death). Relative OR England vs. Scotland

	Elective		Emergency	
**1997/98**	0.890	***	1.027	***
	(0.852-0.930)		(1.012-1.043)	
**1998/99**	0.929	***	1.096	***
	(0.889-0.971)		(1.079-1.113)	
**1999/00**	0.898	***	1.105	***
	(0.859-0.938)		(1.088-1.122)	
**2000/01**	0.952	**	1.105	***
	(0.909-0.996)		(1.088-1.122)	
**2001/02**	0.982		1.121	***
	(0.938-1.029)		(1.104-1.138)	
**2002/03**	0.941	**	1.095	***
	(0.899-0.986)		(1.078-1.111)	
**2003/04**	0.834	***	1.094	***
	(0.797-0.873)		(1.078-1.111)	
**2004/05**	0.776	***	1.069	***
	(0.741-0.813)		(1.053-1.085)	
**2005/06**	0.727	***	1.029	***
	(0.695-0.761)		(1.014-1.045)	
**2006/07**	0.675	***	1.044	***
	(0.644-0.707)		(1.028-1.060)	
**2007/08**	0.560	***	1.002	
	(0.535-0.586)		(0.987-1.017)	
**2008/09**	0.534	***	0.933	***
	(0.511-0.559)		(0.919-0.947)	
**2009/10**	0.511	***	0.868	***
	(0.487-0.536)		(0.855-0.881)	
**2010/11**	0.460	***	0.845	***
	(0.438-0.484)		(0.832-0.858)	
**2011/12**	0.403	***	0.854	***
	(0.383-0.424)		(0.841-0.867)	
**2012/13**	0.370	***	0.827	***
	(0.351-0.390)		(0.815-0.840)	
**2013/14**	0.329	***	0.831	***
	(0.312-0.348)		(0.818-0.844)	
**Dummy Variables:**				
**Age Group**	YES		YES	
**Gender**	YES		YES	
**Derpivation Decile**	YES		YES	
**HRG**	YES		YES	
**Number of Observations**	110,832,641		82,308,205	

Notes: 95% CIs in parenthesis. *** and ** indicate 1% and 5% significance, respectively. All regressions include a constant.

For peer review only

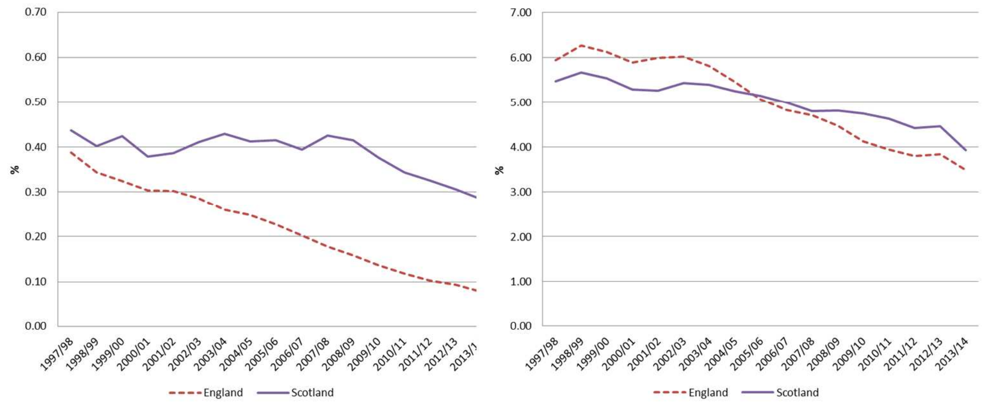

Figure 1. In Hospital Mortality Rate. Elective (LHS) and Emergency (RHS)
Note: the y-axis scales are different; LHS is 1/10 of RHS

170x70mm (257 x 257 DPI)

For peer review only

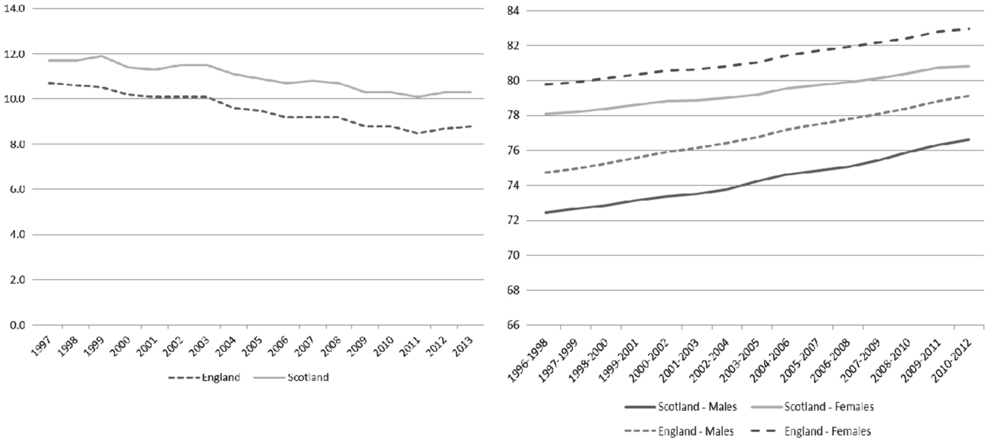

Figure 2. Crude Mortality Rate (LHS) and Life Expectancy (RHS)

180x81mm (200 x 200 DPI)

er review only

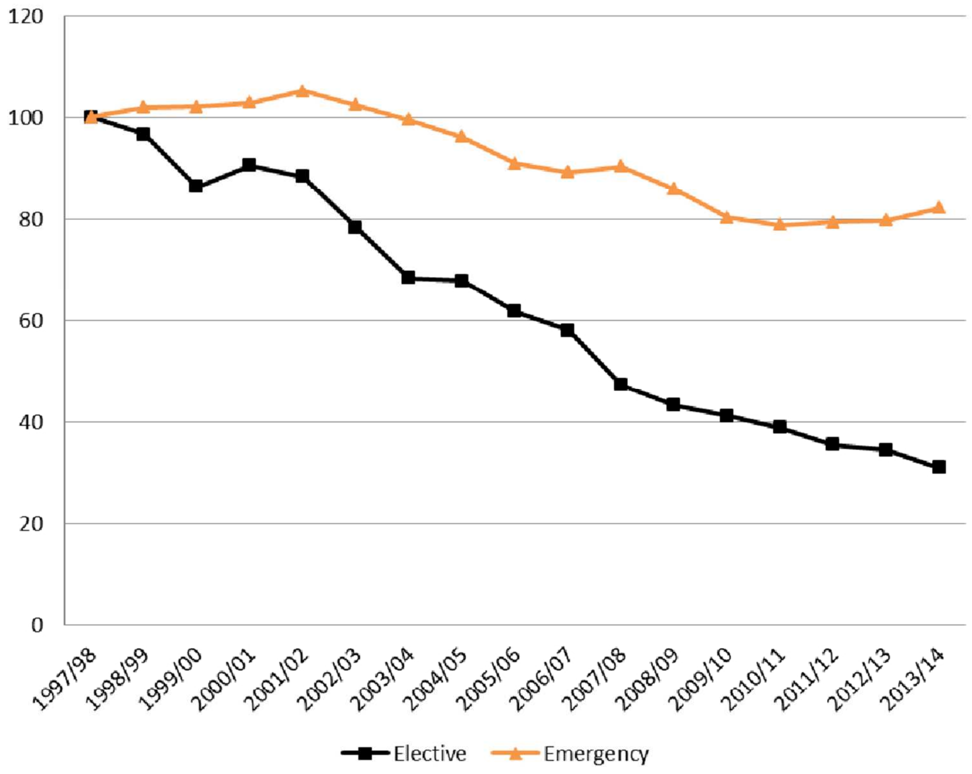

Figure 3. In Hospital Mortality Rate. England/Scotland with 1997/98 = 100

150x120mm (200 x 200 DPI)

BMJ Open

In-hospital mortality is declining faster in England than in Scotland: a regression analysis of hospital admissions over 17 years

Journal:	*BMJ Open*
Manuscript ID	bmjopen-2017-017195.R1
Article Type:	Research
Date Submitted by the Author:	19-May-2017
Complete List of Authors:	Aragon Aragon, Maria Jose; University of York, Centre for Health Economics<br>Chalkley, Martin; University of York, Centre for Health Economics
**Primary Subject Heading**:	Health economics
Secondary Subject Heading:	Health services research
Keywords:	HEALTH ECONOMICS, Health economics < HEALTH SERVICES ADMINISTRATION & MANAGEMENT, Quality in health care < HEALTH SERVICES ADMINISTRATION & MANAGEMENT

SCHOLARONE™
Manuscripts

TITLE PAGE

Title: In-hospital mortality is declining faster in England than in Scotland: a regression analysis of hospital admissions over 17 years.

Authors:

María José Aragón (MJ Aragón) – Corresponding Author
mjma504@york.ac.uk
Research Fellow
Centre for Health Economics, University of York

Martin Chalkley (M Chalkley)
martin.chalkley@york.ac.uk
Professor
Centre for Health Economics, University of York

Centre for Health Economics
Alcuin 'A' Block
University of York
Heslington, York
YO10 5DD UK

Authors' Contributions:
MJ Aragón and M Chalkley defined the research question and type of analysis required, MJ Aragón performed the statistical analysis and MJ Aragón and M Chalkley analysed the results and wrote the article.

Acknowledgements
The Hospital Episode Statistics are copyright © 1997/98 - 2013/14, re-used with the permission of The Health & Social Care Information Centre. All rights reserved.
The Scottish Morbidity Record data was used with the permission of the Information and Statistics Division Scotland (ISD).

For peer review only

Declaration of competing interests

All authors have completed the Unified Competing Interest form at www.icmje.org/coi_disclosure.pdf (available on request from the corresponding author) and Dr. Chalkley and Dr. Aragon have nothing to disclose.

Data Sharing Statement

No additional data available.

Ethics Approval

No ethics approval was required for this study.

Funding Statement

This article was developed further to the NIHR funded project HS&DR - 11/1022/19.

For peer review only

ABSTRACT

Objectives

To examine the trends in in-hospital mortality for England and Scotland over a 17-year period to determine whether and if so to what extent the time trends differ after controlling for differences in the patients treated.

Design

Analysis of retrospective administrative hospital data using descriptive aggregate statistics of trends in in-hospital mortality and estimates of a logistic regression model of individual patient-level in-hospital mortality accounting for patient characteristics, case-mix and country and year specific intercepts.

Setting

Secondary care across all hospitals in England and Scotland from 1997 to 2013.

Population

Over 190 million inpatient admissions, either electively or emergency, in England or Scotland from 1997 to 2013

Data

Hospital Episode Statistics (HES) for England and the Scottish Morbidity Record 01 (SMR) for Scotland.

Main outcome measures

Separately for two admission pathways (elective and emergency) we examine aggregate time trends of the proportion of patients who die in hospital and a binary variable indicating whether an individual patient died in hospital or survived and how that indicator is influenced by the patient's characteristics, the year and the country (England or Scotland) in which they were admitted.

Results

In-hospital mortality has declined in both countries over the period studied, for both elective and emergency admissions but has declined more in England than Scotland. The difference in trend reduction is greater for elective admissions. These differences persist after controlling for patient characteristics and case-mix.

Conclusions

Comparing data at country level suggests questions about the roles performed by or functioning of their health care systems. We found substantial differences between Scotland and England in regard to the trend reductions in in-hospital mortality. Hospital resources are therefore being deployed increasingly differently over time in these two countries for reasons that have yet to be explained.

For peer review only

STRENGTHS AND LIMITATIONS OF THIS STUDY

- The first study to use comprehensive and extensive data on hospital admissions and discharges over a long period of time to study differences in in hospital mortality.
- Establishes a different perspective on in-hospital mortality – that of variation across health care systems over time and establishes that two neighbouring countries with otherwise similar health care systems have different time paths of in-hospital mortality.
- Uses detailed administrative records to control for variation in case-mix and patient characteristics.
- It is not possible to establish the potential causes of the different trends in in-hospital reported but potential causes are established as future avenues of research.

For peer review only

INTRODUCTION

In-hospital mortality has attracted a good deal of attention and concern when used as a proxy for hospital performance (1-6). The concern stems from an inability to disentangle consequences of treatment choices from the inherently different risks that patients' medical conditions pose (7-10). This debate however distracts from the potential knowledge that can be derived by studying in-hospital mortality at a more aggregate level (11). Hospital care is costly and a key resource in addressing a population's health care needs. It has been noted that death is a "core business" of hospitals (12) and hence understanding how that core business is changing – how much of the "business" of hospitals it accounts for – is a crucial aspect of health system planning and management.

Without some reference point it is impossible to determine whether an outcome such as declining in-hospital mortality is notable, or to be expected. Comparing two otherwise similar health care systems establishes each as a reference for the other. That reference is more powerful if analysis is conducted in trends. Differences in the levels of in-hospital mortality across different jurisdictions could easily be accounted for as the consequence of unobserved differences between their populations, health care needs and service organisation. However, these unobservable factors seem likely to follow common trends, so divergence in the trends of in-hospital mortality are more challenging to explain.

This, the first study of its kind, examines the trends in in-hospital mortality for England and Scotland over a 17-year period. We establish that "death as the core business of hospitals" has been declining faster in England than in Scotland over that period.

For peer review only

METHODS

Data

In both England and Scotland data are routinely collected on hospital inpatient activity through, respectively, Hospital Episode Statistics (HES) and the Scottish Morbidity Record (SMR). Both data sources report in terms of episodes (period under the care of one consultant), which are then converted into continuous inpatient spells (CIS) corresponding to the period of care that can include transfers within and between hospitals. We construct equivalent measures of CIS for both countries and distinguish between elective (including day cases) and emergency admissions, excluding maternity and regular attenders, using the type of admission of the first episode in the CIS. Both data sources report on the basis of financial years (1 April to 31 March) but for convenience we denote the financial year by its first calendar year. We examine over 190 million CIS from 1997 to 2013 using discharge information to determine whether the patient died in hospital or not.

Both data sources include the characteristics of a patient in regard to age, sex and the deprivations decile of their home address. We use these together with the Healthcare Resource Group (HRG) into which the patient's treatment fell to account for variation in case-mix.

Empirical Methods

The proportion of all CIS that end in death was calculated directly from the data sources, separately for each country, year and admission pathway.

After constructing a binary outcome variable (equal to 1 if the patient died in hospital and 0 otherwise) logistic regression analysis, separately for each admission pathway, was used to determine whether differences across jurisdictions persist after including covariates. To control for the potential influences of patient characteristics, case-mix and socio-economic circumstances the covariates included in the analysis were age (using five-year age bands indicators), sex (as an indicator equal to one for females), HRG indicators (there are more than 1000 different HRGs in the data) and deprivation decile indicators (1 being the most deprived and 10 the least deprived). Differences between countries were captured by country specific dummy variables and interactions between those and year dummy variables.

We ran logit regressions using Stata13.

For peer review only

RESULTS

Figure 1 shows the trend in the in-hospital mortality (CIS where patient died / Total CIS) for England and Scotland for elective and emergency admissions.

[FIGURE 1 HERE]

In Figure 1 it is apparent that in-hospital mortality has decreased in both countries, but has done so more quickly in England than in Scotland in both emergency and elective care. Over the same period the trends of overall mortality, measured by the crude mortality rate (deaths per 1,000 population), and life expectancy (in years) have been similar in both countries (13-15), see Figure 2. Whilst overall spending per head on hospital care is higher in Scotland it has followed a similar (increasing) trend as in England (16).

[FIGURE 2 HERE]

Next we describe the relative, England/Scotland, in-hospital mortality rate (CIS where patient died / Total CIS), again separately for Elective and Emergency admissions. Figure 3 shows the ratio of the in-hospital mortality rates for both countries, normalising to 100 the initial year, and clearly shows the relative change in the in-hospital mortality rates.

[FIGURE 3 HERE]

Next we determine whether these crude, unadjusted differences persist once we account for the different characteristics of the patients that are being treated in the two countries, specifically; age, sex, disease proxies and deprivation level.

Table 1 shows the descriptive statistics of the variables used for the regression analysis. Over the period of analysis in-hospital mortality has been higher for emergency than for elective admissions, that emergency admissions' patients are more likely to be males, are younger, and more likely to come from the highest deprivation decile than elective admissions.

[TABLE 1 HERE]

Table 2 shows regression results, presented as relative odd ratios between England and Scotland, this presentation was chosen to simplify the results table and focus on the question of interest: is the reduction in in-hospital mortality rates different between the countries after controlling for patient and CIS characteristics?

[TABLE 2 HERE]

The results in Table 2 confirm what is observed in Figures 1 and 3, the reduction in in-hospital mortality in England has been faster than that of Scotland throughout the period, even after controlling for patient and CIS characteristics. The results are reported as odd-ratios, showing the relative difference between the two countries in each period, e.g. the first row says that in the initial year of the analysis Elective admissions were 11% lower in England than in Scotland and Emergency admissions were 3% higher. For *Electives* England starts with a lower in-hospital mortality rate (coefficient in first row is less than one and significant), then there is no clear trend in the difference between the countries until there is no significant difference between the two countries (non-significant coefficient in 2001/02) and then the difference with Scotland increases over time

For peer review only

(coefficient becomes smaller over time) until in the last year in-hospital mortality for Elective admissions in England is around one third of that in Scotland. For *Emergencies* England started with a higher in-hospital mortality rate (coefficient in top row is greater than one and significant) and the difference between the two countries first increased (coefficients become greater) and then decreased until there is no difference between them (non-significant coefficient in 2007/08) and then England's in-hospital mortality rate continues to reduce relative to that of Scotland (coefficients smaller than one and significant from 2008/09 onwards) until being around 27% lower in the last year.

For peer review only

DISCUSSION

This study shows that in-hospital mortality for both elective and emergency admissions has been on a 17-year declining trend in both England and Scotland but that trend reduction has been greater in England. This remains the case after controlling for case mix and population characteristics.

We have used comprehensive and extensive data on hospital admissions and discharges over a long period of time, providing details of more than 190 million admissions. These data have been adjusted so as to be able to compare two similar health care systems so that each can act as a benchmark for the other. Whilst we can establish the differences between the experiences of these two systems with our data, we have not established causal mechanisms for these differences.

Numerous previous studies have examined the variation of in-hospital mortality across different hospitals, focusing on the details and limitations of risk-adjustment. This study provides a different perspective – that of variation across health care systems. Whilst we cannot hope to replicate the detail or depth of previous studies that focus on particular treatments we do provide a much broader and comprehensive view.

That view suggests a number of important and unanswered questions that have great potential importance for policymakers. Why has the divergence in trend reduction in in-hospital mortality developed? In what ways are these two health care systems developing different roles for their hospitals? Should there be a concern in Scotland that in-hospital mortality is decreasing less slowly and is not substantially higher than in its near neighbour England?

Our study cannot answer these questions – that is for future research – but we can give some insight and some clues as to the possible answers. One key difference in the development of hospital-based health care in Scotland and England over the period studied has been the reform of financing undertaken in England. This has been shown to have resulted in an expansion of activity on a per-capita basis (refs). That suggests that part of the explanation for what we have observed is that hospitals in England are treating more "less-sick" patients which would result in a lower propensity for patients to die in hospital simply by increasing the denominator. However, that seems unlikely to be the whole explanation because we have established that the reductions in mortality exist for *both* elective and emergency admissions and whereas the former would appear susceptible to "denominator" effect it is less easy to account for emergency admissions in this way. The reduction on in-hospital mortality could also be related to the reduction in the duration of hospital admissions (usually called 'length of stay', LoS) which both countries report in the period of analysis (17, 18). We use HRGs to adjust for case-mix, however HRGs are meant not only to group together patients with similar diagnosis/treatment but also with similar resource intensity (19). Since we adjusted for the kinds of treatments that are carried out, and the resources needed to deliver them, in England and Scotland it is also difficult to account for the differences in trends in terms of changing case-mix unless our adjustment is substantially flawed because there are large unobserved differences. Basing an analysis on trends mitigates this risk because for it to affect our results requires that the unobserved differences in case mix between the two systems are changing over time.

This then suggests that there are two avenues to explore further. The first is to determine whether the alternatives to care in hospital setting have diverged in the two countries. If for example alternative settings to which terminally ill patients can be discharged have expanded faster in England than in Scotland we would observe the kind of differential trend of in-hospital mortality established by our analysis. The second, more worrying possibility is that there remains some

For peer review only

element of the difference in trend that relates to the efficacy of hospital treatments in the two countries. The details of such potential *quality of care* differences are for clinicians and practitioners who are familiar with hospital treatments of specific conditions to explore, considering any changes in practice or performance targets relevant to them, e.g. during the period of analysis Scotland had targets regarding access and treatment of specific patient groups (20).

For peer review only

REFERENCES

1. Coombes R. Experts disagree about usefulness of hospital mortality data. BMJ : British Medical Journal. 2014;349.

2. Holland W, McKee M. Can hospital mortality comparisons ever be fit for purpose? BMJ : British Medical Journal. 2013;347.

3. Lilford R, Pronovost P. Using hospital mortality rates to judge hospital performance: a bad idea that just won't go away. BMJ. 2010;340.

4. Hogan H, Zipfel R, Neuburger J, Hutchings A, Darzi A, Black N. Avoidability of hospital deaths and association with hospital-wide mortality ratios: retrospective case record review and regression analysis. BMJ : British Medical Journal. 2015;351.

5. Black N. Assessing the quality of hospitals. BMJ. 2010;340.

6. van Gestel YR, Lemmens VE, Lingsma HF, de Hingh IH, Rutten HJ, Coebergh JW. The Hospital Standardized Mortality Ratio Fallacy: A Narrative Review. Medical Care. 2012;50(8):662–7.

7. Girling AJ, Hofer TP, Wu J, Chilton PJ, Nicholl JP, Mohammed MA, et al. Case-mix adjusted hospital mortality is a poor proxy for preventable mortality: a modelling study. BMJ Quality & Safety. 2012;21:1052-6.

8. Stewart K, Choudry MI, Buckingham R. Learning from hospital mortality. Clinical Medicine. 2016;16(6):530-4.

9. Hawkes N. Patient coding and the ratings game. BMJ. 2010;340.

10. Bottle A, Jarman B, Aylin P. Hospital Standardized Mortality Ratios: Sensitivity Analyses on the Impact of Coding. Health Services Research. 2011;46(6pt1):1741-61.

11. Farrar S, Yi D, Sutton M, Chalkley M, Sussex J, Scott A. Has payment by results affected the way that English hospitals provide care? Difference-in-differences analysis. BMJ. 2009;339.

12. Bradford E. Death is 'core business' of Scottish hospitals, study finds. BBC News19 March 2014.

13. Office for National Statistics. Vital Statistics: Population and Health Reference Tables.

14. Office for National Statistics. Life Expectancy at Birth and at Age 65 by Local Areas in England and Wales.

15. Office for National Statistics. Life Expectancy at Birth and at Age 65 for the UK and Local Areas in Scotland.

16. Hawe E, Cockcroft L. OHE Guide to UK Health and Health Care Statistics. 2013.

17. Health and Social Care Information Centre. Hospital Episode Statistics, Admitted Patient Care, England - 2013-14. 2015.

18. Information Services Division. Acute Hospital Activity and NHS Beds Information, Quarter ending March 2014 2014.

For peer review only

19. NHS Digital. Introduction to Healthcare Resource Groups.

20. NHSScotland Performance and Business Management. HEAT Targets due for delivery 2006-2015. 2014.

For peer review only

FIGURES

Figure 1. In Hospital Mortality Rate. Elective (LHS) and Emergency (RHS)
Note: the y-axis scales are different; LHS is 1/10 of RHS.

Figure 2. Crude Mortality Rate (LHS) and Life Expectancy (RHS)

Figure 3. In Hospital Mortality Rate. England/Scotland with 1997/98 = 100.

For peer review only

TABLES

Table 1. Descriptive Statistics

	Mean	Std. Dev.	Mean	Std. Dev.
Elective	**England**		**Scotland**	
**% Died in Hospital**	0.21	4.62	0.40	6.32
**% Males**	46.86	49.90	45.31	49.78
**Age**	54.43	21.26	53.73	21.03
**% Decile 1**	10.26	30.35	12.06	32.57
**% Decile 10**	9.00	28.62	8.21	27.46
**Number of Observations**	100,945,785		9,886,856	
Emergency	**England**		**Scotland**	
**% Died in Hospital**	4.98	21.75	5.01	21.82
**% Males**	48.03	50.00	49.14	49.99
**Age**	50.55	28.32	52.60	26.39
**% Decile 1**	14.70	35.41	15.85	36.52
**% Decile 10**	7.11	25.69	6.21	24.13
**Number of Observations**	74,048,633		8,259,572	

For peer review only

Table 2. Logit Regression Results. Dependent Variable: Prob(Death). Relative OR England vs. Scotland

	Elective		Emergency	
**1997/98**	0.890	***	1.027	***
	(0.852-0.930)		(1.012-1.043)	
**1998/99**	0.929	***	1.096	***
	(0.889-0.971)		(1.079-1.113)	
**1999/00**	0.898	***	1.105	***
	(0.859-0.938)		(1.088-1.122)	
**2000/01**	0.952	**	1.105	***
	(0.909-0.996)		(1.088-1.122)	
**2001/02**	0.982		1.121	***
	(0.938-1.029)		(1.104-1.138)	
**2002/03**	0.941	**	1.095	***
	(0.899-0.986)		(1.078-1.111)	
**2003/04**	0.834	***	1.094	***
	(0.797-0.873)		(1.078-1.111)	
**2004/05**	0.776	***	1.069	***
	(0.741-0.813)		(1.053-1.085)	
**2005/06**	0.727	***	1.029	***
	(0.695-0.761)		(1.014-1.045)	
**2006/07**	0.675	***	1.044	***
	(0.644-0.707)		(1.028-1.060)	
**2007/08**	0.560	***	1.002	
	(0.535-0.586)		(0.987-1.017)	
**2008/09**	0.534	***	0.933	***
	(0.511-0.559)		(0.919-0.947)	
**2009/10**	0.511	***	0.868	***
	(0.487-0.536)		(0.855-0.881)	
**2010/11**	0.460	***	0.845	***
	(0.438-0.484)		(0.832-0.858)	
**2011/12**	0.403	***	0.854	***
	(0.383-0.424)		(0.841-0.867)	
**2012/13**	0.370	***	0.827	***
	(0.351-0.390)		(0.815-0.840)	
**2013/14**	0.329	***	0.831	***
	(0.312-0.348)		(0.818-0.844)	
**Dummy Variables Included as Controls:**				
**Age Group**	YES		YES	
**Gender**	YES		YES	
**Derpivation Decile**	YES		YES	
**HRG**	YES		YES	
**Number of Observations**	110,832,641		82,308,205	

Notes: 95% CIs in parenthesis. *** and ** indicate 1% and 5% significance, respectively. All regressions include a constant.

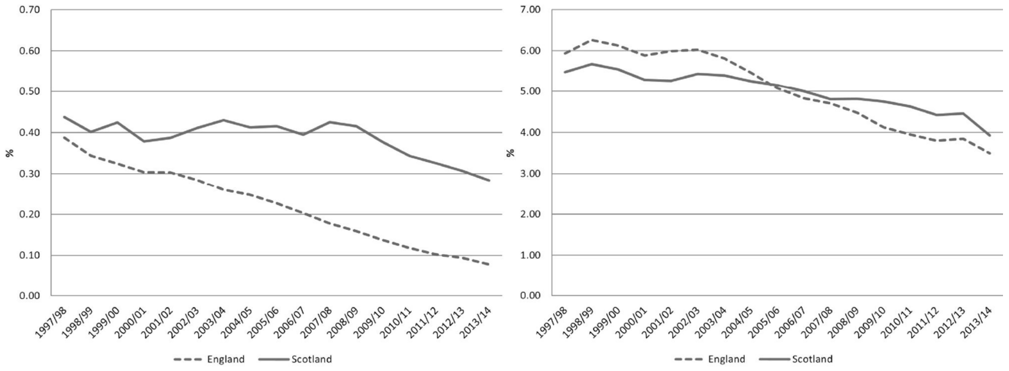

Figure 1. In Hospital Mortality Rate. Elective (LHS) and Emergency (RHS)
Note: the y-axis scales are different; LHS is 1/10 of RHS.

170x66mm (300 x 300 DPI)

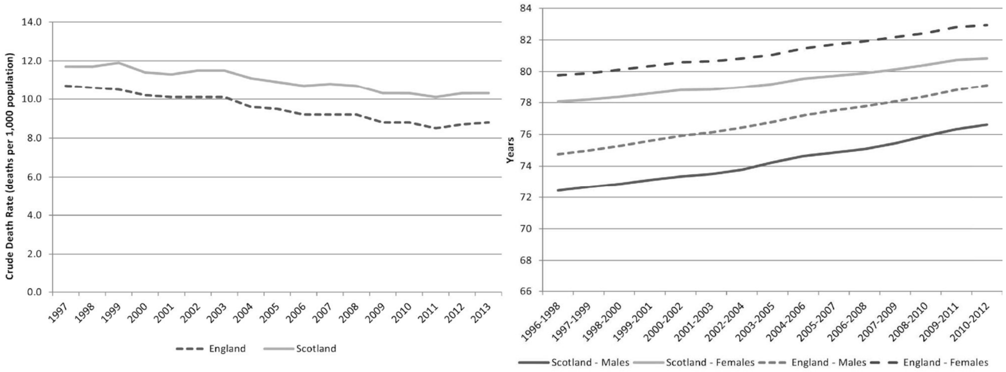

Figure 2. Crude Mortality Rate (LHS) and Life Expectancy (RHS)

170x66mm (300 x 300 DPI)

For peer review only

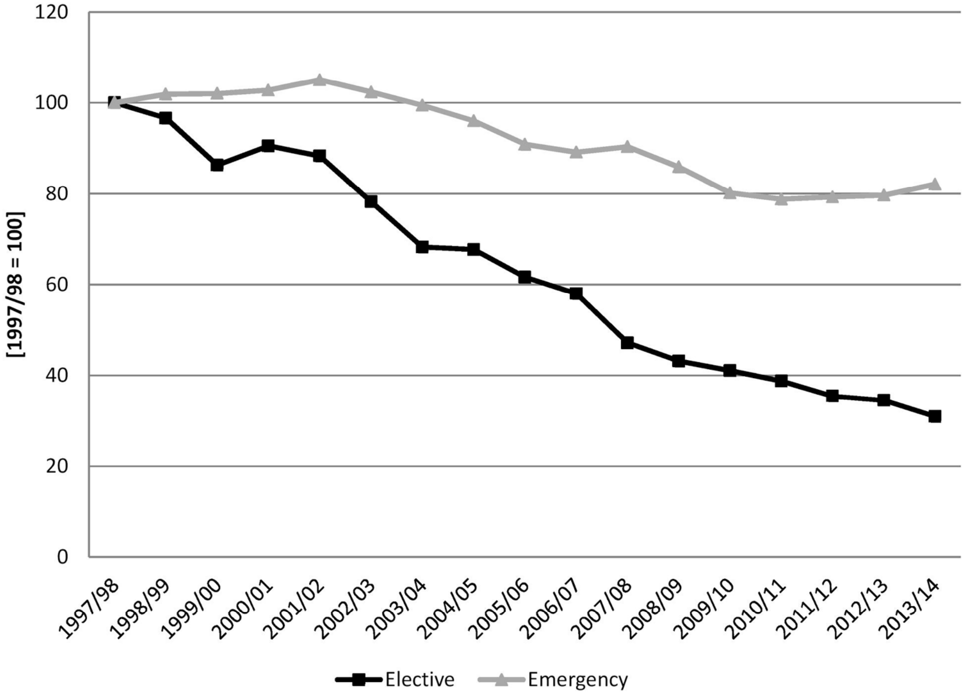

Figure 3. In Hospital Mortality Rate. England/Scotland with 1997/98 = 100

170x126mm (300 x 300 DPI)

BMJ Open

In-hospital mortality is declining faster in England than in Scotland: a regression analysis of hospital admissions over 17 years

Journal:	*BMJ Open*
Manuscript ID	bmjopen-2017-017195.R2
Article Type:	Research
Date Submitted by the Author:	19-Jun-2017
Complete List of Authors:	Aragon Aragon, Maria Jose; University of York, Centre for Health Economics<br>Chalkley, Martin; University of York, Centre for Health Economics
**Primary Subject Heading**:	Health economics
Secondary Subject Heading:	Health services research
Keywords:	HEALTH ECONOMICS, Health economics < HEALTH SERVICES ADMINISTRATION & MANAGEMENT, Quality in health care < HEALTH SERVICES ADMINISTRATION & MANAGEMENT

SCHOLARONE™
Manuscripts

TITLE PAGE

Title: In-hospital mortality is declining faster in England than in Scotland: a regression analysis of hospital admissions over 17 years.

Authors:

María José Aragón (MJ Aragón) – Corresponding Author
mjma504@york.ac.uk
Research Fellow
Centre for Health Economics, University of York

Martin Chalkley (M Chalkley)
martin.chalkley@york.ac.uk
Professor
Centre for Health Economics, University of York

Centre for Health Economics
Alcuin 'A' Block
University of York
Heslington, York
YO10 5DD UK

Authors' Contributions:
MJ Aragón and M Chalkley defined the research question and type of analysis required, MJ Aragón performed the statistical analysis and MJ Aragón and M Chalkley analysed the results and wrote the article.

Acknowledgements
The Hospital Episode Statistics are copyright © 1997/98 - 2013/14, re-used with the permission of The Health & Social Care Information Centre. All rights reserved.
The Scottish Morbidity Record data was used with the permission of the Information and Statistics Division Scotland (ISD).

For peer review only

Declaration of competing interests

All authors have completed the Unified Competing Interest form at www.icmje.org/coi_disclosure.pdf (available on request from the corresponding author) and Dr. Chalkley and Dr. Aragon have nothing to disclose.

Data Sharing Statement

No additional data available.

Ethics Approval

No ethics approval was required for this study.

Funding Statement

This article was developed further to the NIHR funded project HS&DR - 11/1022/19.

For peer review only

ABSTRACT

Objectives

To examine the trends in in-hospital mortality for England and Scotland over a 17-year period to determine whether and if so to what extent the time trends differ after controlling for differences in the patients treated.

Design

Analysis of retrospective administrative hospital data using descriptive aggregate statistics of trends in in-hospital mortality and estimates of a logistic regression model of individual patient-level in-hospital mortality accounting for patient characteristics, case-mix and country and year specific intercepts.

Setting

Secondary care across all hospitals in England and Scotland from 1997 to 2013.

Population

Over 190 million inpatient admissions, either electively or emergency, in England or Scotland from 1997 to 2013

Data

Hospital Episode Statistics (HES) for England and the Scottish Morbidity Record 01 (SMR) for Scotland.

Main outcome measures

Separately for two admission pathways (elective and emergency) we examine aggregate time trends of the proportion of patients who die in hospital and a binary variable indicating whether an individual patient died in hospital or survived and how that indicator is influenced by the patient's characteristics, the year and the country (England or Scotland) in which they were admitted.

Results

In-hospital mortality has declined in both countries over the period studied, for both elective and emergency admissions but has declined more in England than Scotland. The difference in trend reduction is greater for elective admissions. These differences persist after controlling for patient characteristics and case-mix.

Conclusions

Comparing data at country level suggests questions about the roles performed by or functioning of their health care systems. We found substantial differences between Scotland and England in regard to the trend reductions in in-hospital mortality. Hospital resources are therefore being deployed increasingly differently over time in these two countries for reasons that have yet to be explained.

For peer review only

STRENGTHS AND LIMITATIONS OF THIS STUDY

- The first study to use comprehensive and extensive data on hospital admissions and discharges over a long period of time to study differences in in hospital mortality.
- Establishes a different perspective on in-hospital mortality – that of variation across health care systems over time and establishes that two neighbouring countries with otherwise similar health care systems have different time paths of in-hospital mortality.
- Uses detailed administrative records to control for variation in case-mix and patient characteristics.
- It is not possible to establish the potential causes of the different trends in in-hospital reported but potential causes are established as future avenues of research.

For peer review only

INTRODUCTION

In-hospital mortality has attracted a good deal of attention and concern when used as a proxy for hospital performance (1-6). The concern stems from an inability to disentangle consequences of treatment choices from the inherently different risks that patients' medical conditions pose (7-10). This debate however distracts from the potential knowledge that can be derived by studying in-hospital mortality at a more aggregate level (11). Hospital care is costly and a key resource in addressing a population's health care needs. It has been noted that death is a "core business" of hospitals (12) and hence understanding how that core business is changing – how much of the "business" of hospitals it accounts for – is a crucial aspect of health system planning and management.

Without some reference point it is impossible to determine whether an outcome such as declining in-hospital mortality is notable, or to be expected. Comparing two otherwise similar health care systems establishes each as a reference for the other. That reference is more powerful if analysis is conducted in trends. Differences in the levels of in-hospital mortality across different jurisdictions could easily be accounted for as the consequence of unobserved differences between their populations, health care needs and service organisation. However, these unobservable factors seem likely to follow common trends, so divergence in the trends of in-hospital mortality are more challenging to explain.

This, the first study of its kind, examines the trends in in-hospital mortality for England and Scotland over a 17-year period. We establish that "death as the core business of hospitals" has been declining faster in England than in Scotland over that period.

For peer review only

METHODS

Data

In both England and Scotland data are routinely collected on hospital inpatient activity through, respectively, Hospital Episode Statistics (HES) and the Scottish Morbidity Record (SMR). Both data sources report in terms of episodes (period under the care of one consultant), which are then converted into continuous inpatient spells (CIS) corresponding to the period of care that can include transfers within and between hospitals. We construct equivalent measures of CIS for both countries and distinguish between elective (including day cases) and emergency admissions, excluding maternity and regular attenders, using the type of admission of the first episode in the CIS. Both data sources report on the basis of financial years (1 April to 31 March) but for convenience we denote the financial year by its first calendar year. We examine over 190 million CIS from 1997 to 2013 using discharge information to determine whether the patient died in hospital or not.

Both data sources include the characteristics of a patient in regard to age, sex and the deprivations decile of their home address. We use these together with the Healthcare Resource Group (HRG) into which the patient’s treatment fell to account for variation in case-mix.

Empirical Methods

The proportion of all CIS that end in death was calculated directly from the data sources, separately for each country, year and admission pathway.

After constructing a binary outcome variable (equal to 1 if the patient died in hospital and 0 otherwise) logistic regression analysis, separately for each admission pathway, was used to determine whether differences across jurisdictions persist after including covariates. To control for the potential influences of patient characteristics, case-mix and socio-economic circumstances the covariates included in the analysis were age (using five-year age bands indicators), sex (as an indicator equal to one for females), HRG indicators (there are more than 1000 different HRGs in the data) and deprivation decile indicators (1 being the most deprived and 10 the least deprived). Differences between countries were captured by country specific dummy variables and interactions between those and year dummy variables.

We ran logit regressions using Stata13.

For peer review only

RESULTS

Figure 1 shows the trend in the in-hospital mortality (CIS where patient died / Total CIS) for England and Scotland for elective and emergency admissions.

[FIGURE 1 HERE]

In Figure 1 it is apparent that in-hospital mortality has decreased in both countries, but has done so more quickly in England than in Scotland in both emergency and elective care. Over the same period the trends of overall mortality, measured by the crude mortality rate (deaths per 1,000 population), and life expectancy (in years) have been similar in both countries (13-15), see Figure 2. Whilst overall spending per head on hospital care is higher in Scotland it has followed a similar (increasing) trend as in England (16).

[FIGURE 2 HERE]

Next we describe the relative, England/Scotland, in-hospital mortality rate (CIS where patient died / Total CIS), again separately for Elective and Emergency admissions. Figure 3 shows the ratio of the in-hospital mortality rates for both countries, normalising to 100 the initial year, and clearly shows the relative change in the in-hospital mortality rates.

[FIGURE 3 HERE]

Next we determine whether these crude, unadjusted differences persist once we account for the different characteristics of the patients that are being treated in the two countries, specifically; age, sex, disease proxies and deprivation level.

Table 1 shows the descriptive statistics of the variables used for the regression analysis. Over the period of analysis in-hospital mortality has been higher for emergency than for elective admissions, that emergency admissions' patients are more likely to be males, are younger, and more likely to come from the highest deprivation decile than elective admissions.

[TABLE 1 HERE]

Table 2 shows regression results, presented as relative odd ratios between England and Scotland, this presentation was chosen to simplify the results table and focus on the question of interest: is the reduction in in-hospital mortality rates different between the countries after controlling for patient and CIS characteristics?

[TABLE 2 HERE]

The results in Table 2 confirm what is observed in Figures 1 and 3, the reduction in in-hospital mortality in England has been faster than that of Scotland throughout the period, even after controlling for patient and CIS characteristics. The results are reported as odd-ratios, showing the relative difference between the two countries in each period, e.g. the first row says that in the initial year of the analysis Elective admissions were 11% lower in England than in Scotland and Emergency admissions were 3% higher. For *Electives* England starts with a lower in-hospital mortality rate (coefficient in first row is less than one and significant), then there is no clear trend in the difference between the countries until there is no significant difference between the two countries (non-significant coefficient in 2001/02) and then the difference with Scotland increases over time

For peer review only

(coefficient becomes smaller over time) until in the last year in-hospital mortality for Elective admissions in England is around one third of that in Scotland. For *Emergencies* England started with a higher in-hospital mortality rate (coefficient in top row is greater than one and significant) and the difference between the two countries first increased (coefficients become greater) and then decreased until there is no difference between them (non-significant coefficient in 2007/08) and then England’s in-hospital mortality rate continues to reduce relative to that of Scotland (coefficients smaller than one and significant from 2008/09 onwards) until being around 27% lower in the last year.

As it can be expected for a study comparing two specific countries, these results are not readily generalisable; the comparison of two specific countries, with similar health care systems, will yield a set of results which may or may not correspond to those obtained by comparing any other pair of countries. However, we have established a method of comparison which can apply in any circumstances in which there are suitable data.

For peer review only

DISCUSSION

This study shows that in-hospital mortality for both elective and emergency admissions has been on a 17-year declining trend in both England and Scotland but that trend reduction has been greater in England. This remains the case after controlling for case mix and population characteristics.

We have used comprehensive and extensive data on hospital admissions and discharges over a long period of time, providing details of more than 190 million admissions. These data have been adjusted so as to be able to compare two similar health care systems so that each can act as a benchmark for the other. Whilst we can establish the differences between the experiences of these two systems with our data, we have not established causal mechanisms for these differences.

Numerous previous studies have examined the variation of in-hospital mortality across different hospitals, focusing on the details and limitations of risk-adjustment. This study provides a different perspective – that of variation across health care systems. Whilst we cannot hope to replicate the detail or depth of previous studies that focus on particular treatments we do provide a much broader and comprehensive view.

That view suggests a number of important and unanswered questions that have great potential importance for policymakers. Why has the divergence in trend reduction in in-hospital mortality developed? In what ways are these two health care systems developing different roles for their hospitals? Should there be a concern in Scotland that in-hospital mortality is decreasing less slowly and is not substantially higher than in its near neighbour England?

Our study cannot answer these questions – that is for future research – but we can give some insight and some clues as to the possible answers. One key difference in the development of hospital-based health care in Scotland and England over the period studied has been the reform of financing undertaken in England. This has been shown to have resulted in an expansion of activity on a per-capita basis (refs). That suggests that part of the explanation for what we have observed is that hospitals in England are treating more "less-sick" patients which would result in a lower propensity for patients to die in hospital simply by increasing the denominator. However, that seems unlikely to be the whole explanation because we have established that the reductions in mortality exist for *both* elective and emergency admissions and whereas the former would appear susceptible to "denominator" effect it is less easy to account for emergency admissions in this way. The reduction on in-hospital mortality could also be related to the reduction in the duration of hospital admissions (usually called 'length of stay', LoS) which both countries report in the period of analysis (17, 18). We use HRGs to adjust for case-mix, however HRGs are meant not only to group together patients with similar diagnosis/treatment but also with similar resource intensity (19). Since we adjusted for the kinds of treatments that are carried out, and the resources needed to deliver them, in England and Scotland it is also difficult to account for the differences in trends in terms of changing case-mix unless our adjustment is substantially flawed because there are large unobserved differences. Basing an analysis on trends mitigates this risk because for it to affect our results requires that the unobserved differences in case mix between the two systems are changing over time.

This then suggests that there are two avenues to explore further. The first is to determine whether the alternatives to care in hospital setting have diverged in the two countries. If for example alternative settings to which terminally ill patients can be discharged have expanded faster in England than in Scotland we would observe the kind of differential trend of in-hospital mortality established by our analysis. The second, more worrying possibility is that there remains some

For peer review only

element of the difference in trend that relates to the efficacy of hospital treatments in the two countries. The details of such potential *quality of care* differences are for clinicians and practitioners who are familiar with hospital treatments of specific conditions to explore, considering any changes in practice or performance targets relevant to them, e.g. during the period of analysis Scotland had targets regarding access and treatment of specific patient groups (20).

For peer review only

REFERENCES

1. Coombes R. Experts disagree about usefulness of hospital mortality data. BMJ : British Medical Journal. 2014;349.

2. Holland W, McKee M. Can hospital mortality comparisons ever be fit for purpose? BMJ : British Medical Journal. 2013;347.

3. Lilford R, Pronovost P. Using hospital mortality rates to judge hospital performance: a bad idea that just won't go away. BMJ. 2010;340.

4. Hogan H, Zipfel R, Neuburger J, Hutchings A, Darzi A, Black N. Avoidability of hospital deaths and association with hospital-wide mortality ratios: retrospective case record review and regression analysis. BMJ : British Medical Journal. 2015;351.

5. Black N. Assessing the quality of hospitals. BMJ. 2010;340.

6. van Gestel YR, Lemmens VE, Lingsma HF, de Hingh IH, Rutten HJ, Coebergh JW. The Hospital Standardized Mortality Ratio Fallacy: A Narrative Review. Medical Care. 2012;50(8):662–7.

7. Girling AJ, Hofer TP, Wu J, Chilton PJ, Nicholl JP, Mohammed MA, et al. Case-mix adjusted hospital mortality is a poor proxy for preventable mortality: a modelling study. BMJ Quality & Safety. 2012;21:1052-6.

8. Stewart K, Choudry MI, Buckingham R. Learning from hospital mortality. Clinical Medicine. 2016;16(6):530-4.

9. Hawkes N. Patient coding and the ratings game. BMJ. 2010;340.

10. Bottle A, Jarman B, Aylin P. Hospital Standardized Mortality Ratios: Sensitivity Analyses on the Impact of Coding. Health Services Research. 2011;46(6pt1):1741-61.

11. Farrar S, Yi D, Sutton M, Chalkley M, Sussex J, Scott A. Has payment by results affected the way that English hospitals provide care? Difference-in-differences analysis. BMJ. 2009;339.

12. Bradford E. Death is 'core business' of Scottish hospitals, study finds. BBC News19 March 2014.

13. Office for National Statistics. Vital Statistics: Population and Health Reference Tables.

14. Office for National Statistics. Life Expectancy at Birth and at Age 65 by Local Areas in England and Wales.

15. Office for National Statistics. Life Expectancy at Birth and at Age 65 for the UK and Local Areas in Scotland.

16. Hawe E, Cockcroft L. OHE Guide to UK Health and Health Care Statistics. 2013.

17. Health and Social Care Information Centre. Hospital Episode Statistics, Admitted Patient Care, England - 2013-14. 2015.

18. Information Services Division. Acute Hospital Activity and NHS Beds Information, Quarter ending March 2014 2014.

For peer review only

19. NHS Digital. Introduction to Healthcare Resource Groups.

20. NHSScotland Performance and Business Management. HEAT Targets due for delivery 2006-2015. 2014.

For peer review only

FIGURES

Figure 1. In Hospital Mortality Rate. Elective (LHS) and Emergency (RHS)
Note: the y-axis scales are different; LHS is 1/10 of RHS.

Figure 2. Crude Mortality Rate (LHS) and Life Expectancy (RHS)

Figure 3. In Hospital Mortality Rate. England/Scotland with 1997/98 = 100.

For peer review only

TABLES

Table 1. Descriptive Statistics

	Mean	Std. Dev.	Mean	Std. Dev.
Elective	**England**		**Scotland**	
**% Died in Hospital**	0.21	4.62	0.40	6.32
**% Males**	46.86	49.90	45.31	49.78
**Age**	54.43	21.26	53.73	21.03
**% Decile 1**	10.26	30.35	12.06	32.57
**% Decile 10**	9.00	28.62	8.21	27.46
**Number of Observations**	100,945,785		9,886,856	
Emergency	**England**		**Scotland**	
**% Died in Hospital**	4.98	21.75	5.01	21.82
**% Males**	48.03	50.00	49.14	49.99
**Age**	50.55	28.32	52.60	26.39
**% Decile 1**	14.70	35.41	15.85	36.52
**% Decile 10**	7.11	25.69	6.21	24.13
**Number of Observations**	74,048,633		8,259,572	

For peer review only

Table 2. Logit Regression Results. Dependent Variable: Prob(Death). Relative OR England vs. Scotland

	Elective	Emergency
**1997/98**	0.890 ***	1.027 ***
	(0.852-0.930)	(1.012-1.043)
**1998/99**	0.929 ***	1.096 ***
	(0.889-0.971)	(1.079-1.113)
**1999/00**	0.898 ***	1.105 ***
	(0.859-0.938)	(1.088-1.122)
**2000/01**	0.952 **	1.105 ***
	(0.909-0.996)	(1.088-1.122)
**2001/02**	0.982	1.121 ***
	(0.938-1.029)	(1.104-1.138)
**2002/03**	0.941 **	1.095 ***
	(0.899-0.986)	(1.078-1.111)
**2003/04**	0.834 ***	1.094 ***
	(0.797-0.873)	(1.078-1.111)
**2004/05**	0.776 ***	1.069 ***
	(0.741-0.813)	(1.053-1.085)
**2005/06**	0.727 ***	1.029 ***
	(0.695-0.761)	(1.014-1.045)
**2006/07**	0.675 ***	1.044 ***
	(0.644-0.707)	(1.028-1.060)
**2007/08**	0.560 ***	1.002
	(0.535-0.586)	(0.987-1.017)
**2008/09**	0.534 ***	0.933 ***
	(0.511-0.559)	(0.919-0.947)
**2009/10**	0.511 ***	0.868 ***
	(0.487-0.536)	(0.855-0.881)
**2010/11**	0.460 ***	0.845 ***
	(0.438-0.484)	(0.832-0.858)
**2011/12**	0.403 ***	0.854 ***
	(0.383-0.424)	(0.841-0.867)
**2012/13**	0.370 ***	0.827 ***
	(0.351-0.390)	(0.815-0.840)
**2013/14**	0.329 ***	0.831 ***
	(0.312-0.348)	(0.818-0.844)
**Dummy Variables Included as Controls:**		
**Age Group**	YES	YES
**Gender**	YES	YES
**Deprivation Decile**	YES	YES
**HRG**	YES	YES
**Number of Observations**	110,832,641	82,308,205

Notes: 95% CIs in parenthesis. *** and ** indicate 1% and 5% significance, respectively. All regressions include a constant.

For peer review only

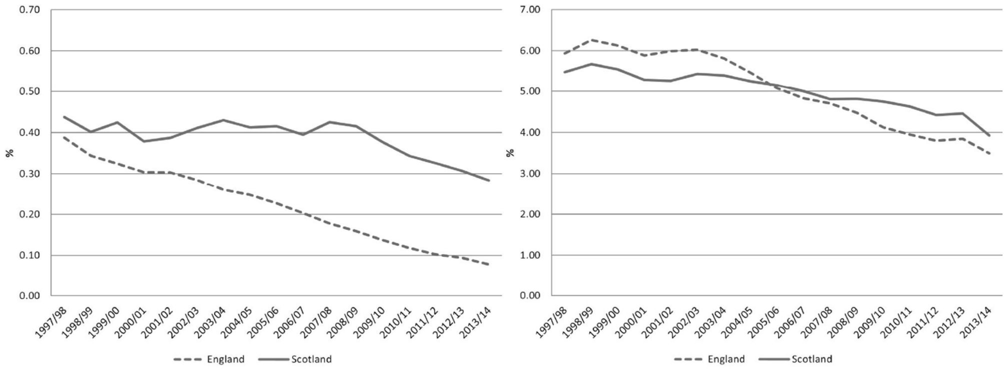

Figure 1. In Hospital Mortality Rate. Elective (LHS) and Emergency (RHS)
Note: the y-axis scales are different; LHS is 1/10 of RHS.

170x66mm (300 x 300 DPI)

For peer review only

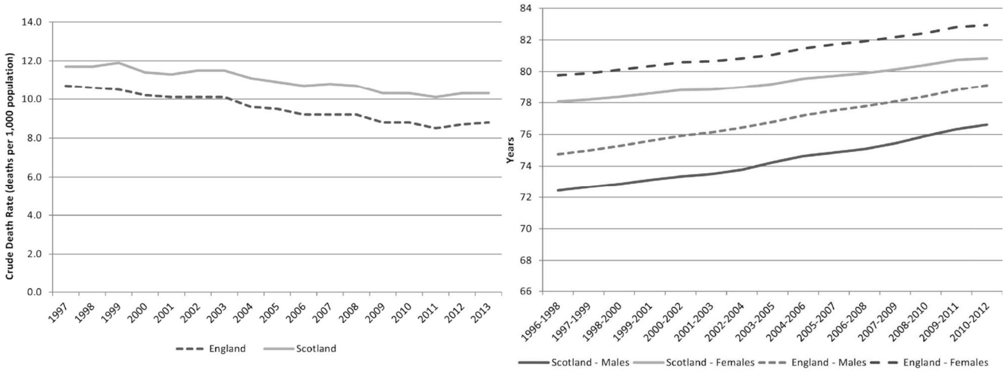

Figure 2. Crude Mortality Rate (LHS) and Life Expectancy (RHS)

170x66mm (300 x 300 DPI)

For peer review only

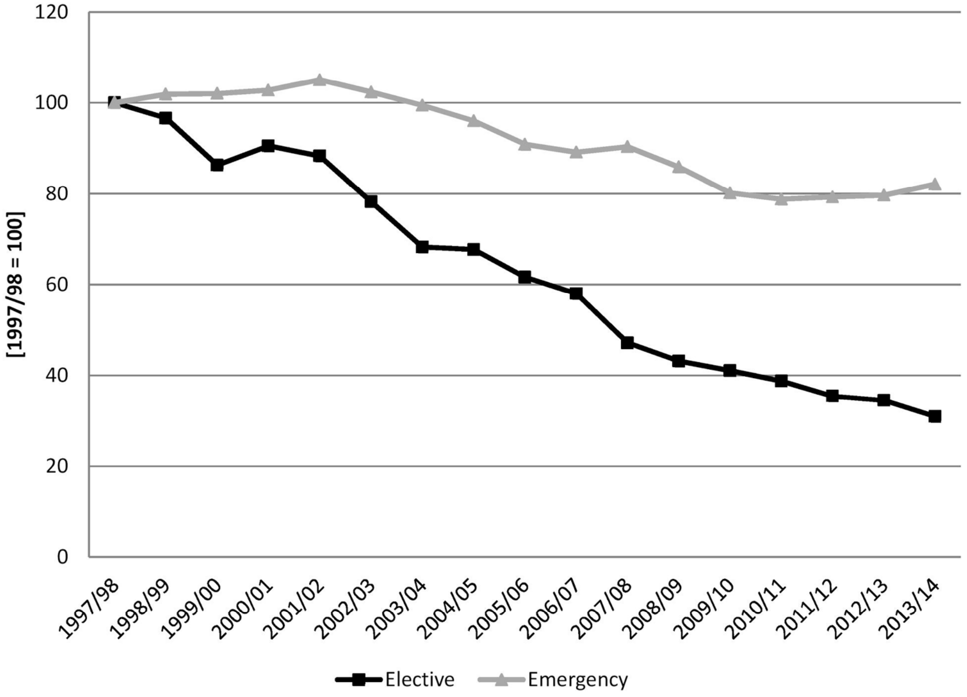

Figure 3. In Hospital Mortality Rate. England/Scotland with 1997/98 = 100

170x126mm (300 x 300 DPI)

BMJ Open

How do time trends in in-hospital mortality compare? A retrospective study of England and Scotland over 17 years using administrative data.

Journal:	*BMJ Open*
Manuscript ID	bmjopen-2017-017195.R3
Article Type:	Research
Date Submitted by the Author:	16-Aug-2017
Complete List of Authors:	Aragon Aragon, Maria Jose; University of York, Centre for Health Economics<br>Chalkley, Martin; University of York, Centre for Health Economics
**Primary Subject Heading**:	Health economics
Secondary Subject Heading:	Health services research
Keywords:	HEALTH ECONOMICS, Health economics < HEALTH SERVICES ADMINISTRATION & MANAGEMENT, Quality in health care < HEALTH SERVICES ADMINISTRATION & MANAGEMENT

SCHOLARONE™
Manuscripts

For peer review only

TITLE PAGE

Title: How do time trends in in-hospital mortality compare? A retrospective study of England and Scotland over 17 years using administrative data.

Authors:

María José Aragón (MJ Aragón) – Corresponding Author
mjma504@york.ac.uk
Research Fellow
Centre for Health Economics, University of York

Martin Chalkley (M Chalkley)
martin.chalkley@york.ac.uk
Professor
Centre for Health Economics, University of York

Centre for Health Economics
Alcuin 'A' Block
University of York
Heslington, York
YO10 5DD UK

Authors' Contributions:

MJ Aragón and M Chalkley defined the research question and type of analysis required, MJ Aragón performed the statistical analysis and MJ Aragón and M Chalkley analysed the results and wrote the article.

Acknowledgements

The Hospital Episode Statistics are copyright © 1997/98 - 2013/14, re-used with the permission of The Health & Social Care Information Centre. All rights reserved.
The Scottish Morbidity Record data was used with the permission of the Information and Statistics Division Scotland (ISD).

For peer review only

Declaration of competing interests

All authors have completed the Unified Competing Interest form at www.icmje.org/coi_disclosure.pdf (available on request from the corresponding author) and Dr. Chalkley and Dr. Aragon have nothing to disclose.

Data Sharing Statement

No additional data available.

Ethics Approval

No ethics approval was required for this study.

Funding Statement

This article was developed further to the NIHR funded project HS&DR - 11/1022/19.

For peer review only

ABSTRACT

Objectives

To examine the trends in in-hospital mortality for England and Scotland over a 17-year period to determine whether and if so to what extent the time trends differ after controlling for differences in the patients treated.

Design

Analysis of retrospective administrative hospital data using descriptive aggregate statistics of trends in in-hospital mortality and estimates of a logistic regression model of individual patient-level in-hospital mortality accounting for patient characteristics, case-mix and country and year specific intercepts.

Setting

Secondary care across all hospitals in England and Scotland from 1997 to 2013.

Population

Over 190 million inpatient admissions, either electively or emergency, in England or Scotland from 1997 to 2013

Data

Hospital Episode Statistics (HES) for England and the Scottish Morbidity Record 01 (SMR) for Scotland.

Main outcome measures

Separately for two admission pathways (elective and emergency) we examine aggregate time trends of the proportion of patients who die in hospital and a binary variable indicating whether an individual patient died in hospital or survived and how that indicator is influenced by the patient's characteristics, the year and the country (England or Scotland) in which they were admitted.

Results

In-hospital mortality has declined in both countries over the period studied, for both elective and emergency admissions but has declined more in England than Scotland. The difference in trend reduction is greater for elective admissions. These differences persist after controlling for patient characteristics and case-mix.

Conclusions

Comparing data at country level suggests questions about the roles performed by or functioning of their health care systems. We found substantial differences between Scotland and England in regard to the trend reductions in in-hospital mortality. Hospital resources are therefore being deployed increasingly differently over time in these two countries for reasons that have yet to be explained.

For peer review only

STRENGTHS AND LIMITATIONS OF THIS STUDY

- The first study to use comprehensive and extensive data on hospital admissions and discharges over a long period of time to study differences in in hospital mortality.
- Establishes a different perspective on in-hospital mortality – that of variation across health care systems over time and establishes that two neighbouring countries with otherwise similar health care systems have different time paths of in-hospital mortality.
- Uses detailed administrative records to control for variation in case-mix and patient characteristics.
- It is not possible to establish the potential causes of the different trends in in-hospital reported but potential causes are established as future avenues of research.

For peer review only

INTRODUCTION

In-hospital mortality has attracted a good deal of attention and concern when used as a proxy for hospital performance (1-6). The concern stems from an inability to disentangle consequences of treatment choices from the inherently different risks that patients' medical conditions pose (7-10). This debate however distracts from the potential knowledge that can be derived by studying in-hospital mortality at a more aggregate level (11). Hospital care is costly and a key resource in addressing a population's health care needs. It has been noted that death is a "core business" of hospitals (12) and hence understanding how that core business is changing – how much of the "business" of hospitals it accounts for – is a crucial aspect of health system planning and management.

Without some reference point it is impossible to determine whether an outcome such as declining in-hospital mortality is notable, or to be expected. Comparing two otherwise similar health care systems establishes each as a reference for the other. That reference is more powerful if analysis is conducted in trends. Differences in the levels of in-hospital mortality across different jurisdictions could easily be accounted for as the consequence of unobserved differences between their populations, health care needs and service organisation. However, these unobservable factors seem likely to follow common trends, so divergence in the trends of in-hospital mortality are more challenging to explain.

This, the first study of its kind, examines the trends in in-hospital mortality for England and Scotland over a 17-year period. We establish that "death as the core business of hospitals" has been declining faster in England than in Scotland over that period.

For peer review only

METHODS

Data

In both England and Scotland data are routinely collected on hospital inpatient activity through, respectively, Hospital Episode Statistics (HES) and the Scottish Morbidity Record (SMR). Both data sources report in terms of episodes (period under the care of one consultant), which are then converted into continuous inpatient spells (CIS) corresponding to the period of care that can include transfers within and between hospitals. We construct equivalent measures of CIS for both countries and distinguish between elective (including day cases) and emergency admissions, excluding maternity and regular attenders, using the type of admission of the first episode in the CIS. Both data sources report on the basis of financial years (1 April to 31 March) but for convenience we denote the financial year by its first calendar year. We examine over 190 million CIS from 1997 to 2013 using discharge information to determine whether the patient died in hospital or not.

Both data sources include the characteristics of a patient in regard to age, sex and the deprivations decile of their home address. We use these together with the Healthcare Resource Group (HRG) into which the patient's treatment fell to account for variation in case-mix.

Empirical Methods

The proportion of all CIS that end in death was calculated directly from the data sources, separately for each country, year and admission pathway.

After constructing a binary outcome variable (equal to 1 if the patient died in hospital and 0 otherwise) logistic regression analysis, separately for each admission pathway, was used to determine whether differences across jurisdictions persist after including covariates. To control for the potential influences of patient characteristics, case-mix and socio-economic circumstances the covariates included in the analysis were age (using five-year age bands indicators), sex (as an indicator equal to one for females), HRG indicators (there are more than 1000 different HRGs in the data) and deprivation decile indicators (1 being the most deprived and 10 the least deprived). Differences between countries were captured by country specific dummy variables and interactions between those and year dummy variables.

We ran logit regressions using Stata13.

For peer review only

RESULTS

Figure 1 shows the trend in the in-hospital mortality (CIS where patient died / Total CIS) for England and Scotland for elective and emergency admissions.

[FIGURE 1 HERE]

In Figure 1 it is apparent that in-hospital mortality has decreased in both countries, but has done so more quickly in England than in Scotland in both emergency and elective care. Over the same period the trends of overall mortality, measured by the crude mortality rate (deaths per 1,000 population), and life expectancy (in years) have been similar in both countries (13-15), see Figure 2. Whilst overall spending per head on hospital care is higher in Scotland it has followed a similar (increasing) trend as in England (16).

[FIGURE 2 HERE]

Next we describe the relative, England/Scotland, in-hospital mortality rate (CIS where patient died / Total CIS), again separately for Elective and Emergency admissions. Figure 3 shows the ratio of the in-hospital mortality rates for both countries, normalising to 100 the initial year, and clearly shows the relative change in the in-hospital mortality rates.

[FIGURE 3 HERE]

Next we determine whether these crude, unadjusted differences persist once we account for the different characteristics of the patients that are being treated in the two countries, specifically; age, sex, disease proxies and deprivation level.

Table 1 shows the descriptive statistics of the variables used for the regression analysis. Over the period of analysis in-hospital mortality has been higher for emergency than for elective admissions, that emergency admissions' patients are more likely to be males, are younger, and more likely to come from the highest deprivation decile than elective admissions.

Table 1. Descriptive Statistics

	Mean	Std. Dev.	Mean	Std. Dev.
Elective	**England**		**Scotland**	
**% Died in Hospital**	0.21	4.62	0.40	6.32
**% Males**	46.86	49.90	45.31	49.78
**Age**	54.43	21.26	53.73	21.03
**% Decile 1**	10.26	30.35	12.06	32.57
**% Decile 10**	9.00	28.62	8.21	27.46
**Number of Observations**	100,945,785		9,886,856	
Emergency	**England**		**Scotland**	
**% Died in Hospital**	4.98	21.75	5.01	21.82
**% Males**	48.03	50.00	49.14	49.99
**Age**	50.55	28.32	52.60	26.39
**% Decile 1**	14.70	35.41	15.85	36.52
**% Decile 10**	7.11	25.69	6.21	24.13
**Number of Observations**	74,048,633		8,259,572	

For peer review only

Table 2 shows regression results, presented as relative odd ratios between England and Scotland, this presentation was chosen to simplify the results table and focus on the question of interest: is the reduction in in-hospital mortality rates different between the countries after controlling for patient and CIS characteristics?

The results in Table 2 confirm what is observed in Figures 1 and 3, the reduction in in-hospital mortality in England has been faster than that of Scotland throughout the period, even after controlling for patient and CIS characteristics. The results are reported as odd-ratios, showing the relative difference between the two countries in each period, e.g. the first row says that in the initial year of the analysis Elective admissions were 11% lower in England than in Scotland and Emergency admissions were 3% higher. For *Electives* England starts with a lower in-hospital mortality rate (coefficient in first row is less than one and significant), then there is no clear trend in the difference between the countries until there is no significant difference between the two countries (non-significant coefficient in 2001/02) and then the difference with Scotland increases over time (coefficient becomes smaller over time) until in the last year in-hospital mortality for Elective admissions in England is around one third of that in Scotland. For *Emergencies* England started with a higher in-hospital mortality rate (coefficient in top row is greater than one and significant) and the difference between the two countries first increased (coefficients become greater) and then decreased until there is no difference between them (non-significant coefficient in 2007/08) and then England's in-hospital mortality rate continues to reduce relative to that of Scotland (coefficients smaller than one and significant from 2008/09 onwards) until being around 27% lower in the last year.

As it can be expected for a study comparing two specific countries, these results are not readily generalisable; the comparison of two specific countries, with similar health care systems, will yield a set of results which may or may not correspond to those obtained by comparing any other pair of countries. However, we have established a method of comparison which can apply in any circumstances in which there are suitable data.

For peer review only

Table 2. Logit Regression Results. Dependent Variable: Prob(Death). Relative OR England vs. Scotland

	Elective		Emergency	
**1997/98**	0.890	***	1.027	***
	(0.852-0.930)		(1.012-1.043)	
**1998/99**	0.929	***	1.096	***
	(0.889-0.971)		(1.079-1.113)	
**1999/00**	0.898	***	1.105	***
	(0.859-0.938)		(1.088-1.122)	
**2000/01**	0.952	**	1.105	***
	(0.909-0.996)		(1.088-1.122)	
**2001/02**	0.982		1.121	***
	(0.938-1.029)		(1.104-1.138)	
**2002/03**	0.941	**	1.095	***
	(0.899-0.986)		(1.078-1.111)	
**2003/04**	0.834	***	1.094	***
	(0.797-0.873)		(1.078-1.111)	
**2004/05**	0.776	***	1.069	***
	(0.741-0.813)		(1.053-1.085)	
**2005/06**	0.727	***	1.029	***
	(0.695-0.761)		(1.014-1.045)	
**2006/07**	0.675	***	1.044	***
	(0.644-0.707)		(1.028-1.060)	
**2007/08**	0.560	***	1.002	
	(0.535-0.586)		(0.987-1.017)	
**2008/09**	0.534	***	0.933	***
	(0.511-0.559)		(0.919-0.947)	
**2009/10**	0.511	***	0.868	***
	(0.487-0.536)		(0.855-0.881)	
**2010/11**	0.460	***	0.845	***
	(0.438-0.484)		(0.832-0.858)	
**2011/12**	0.403	***	0.854	***
	(0.383-0.424)		(0.841-0.867)	
**2012/13**	0.370	***	0.827	***
	(0.351-0.390)		(0.815-0.840)	
**2013/14**	0.329	***	0.831	***
	(0.312-0.348)		(0.818-0.844)	
**Dummy Variables Included as Controls:**				
**Age Group**	YES		YES	
**Gender**	YES		YES	
**Deprivation Decile**	YES		YES	
**HRG**	YES		YES	
**Number of Observations**	110,832,641		82,308,205	

Notes: 95% CIs in parenthesis. *** and ** indicate 1% and 5% significance, respectively. All regressions include a constant.

For peer review only

DISCUSSION

This study shows that in-hospital mortality for both elective and emergency admissions has been on a 17-year declining trend in both England and Scotland but that trend reduction has been greater in England. This remains the case after controlling for case mix and population characteristics.

We have used comprehensive and extensive data on hospital admissions and discharges over a long period of time, providing details of more than 190 million admissions. These data have been adjusted so as to be able to compare two similar health care systems so that each can act as a benchmark for the other. Whilst we can establish the differences between the experiences of these two systems with our data, we have not established causal mechanisms for these differences.

Numerous previous studies have examined the variation of in-hospital mortality across different hospitals, focusing on the details and limitations of risk-adjustment. This study provides a different perspective – that of variation across health care systems. Whilst we cannot hope to replicate the detail or depth of previous studies that focus on particular treatments we do provide a much broader and comprehensive view.

That view suggests a number of important and unanswered questions that have great potential importance for policymakers. Why has the divergence in trend reduction in in-hospital mortality developed? In what ways are these two health care systems developing different roles for their hospitals? Should there be a concern in Scotland that in-hospital mortality is decreasing less slowly and is not substantially higher than in its near neighbour England?

Answering the first of these questions will involve a search for clinical factors that may have exerted a differential impact on in-hospital mortality trends in the two countries. There are a number of candidates for such clinical confounders including, for example, the differential timing of the introduction of screening programs for high mortality conditions such as abdominal aortic aneurysm (17). It is worth noting, however, that any one factor is likely to account for only a small fraction of the difference in aggregate trends.

The subsequent questions concern the impact of health system reform and policies once all clinical factors are accounted for. These are also for future research – but we can give some insight and some clues as to the possible answers. One key difference in the development of hospital-based health care in Scotland and England over the period studied has been the reform of financing undertaken in England. This has been shown to have resulted in an expansion of activity on a per-capita basis. That suggests that part of the explanation for what we have observed is that hospitals in England are treating more "less-sick" patients which would result in a lower propensity for patients to die in hospital simply by increasing the denominator. However, that seems unlikely to be the whole explanation because we have established that the reductions in mortality exist for *both* elective and emergency admissions and whereas the former would appear susceptible to "denominator" effect it is less easy to account for emergency admissions in this way. The reduction on in-hospital mortality could also be related to the reduction in the duration of hospital admissions (usually called 'length of stay', LoS) which both countries report in the period of analysis (18, 19). We use HRGs to adjust for case-mix, however HRGs are meant not only to group together patients with similar diagnosis/treatment but also with similar resource intensity (20). Since we adjusted for the kinds of treatments that are carried out, and the resources needed to deliver them, in England and Scotland it is also difficult to account for the differences in trends in terms of changing case-mix

For peer review only

unless our adjustment is substantially flawed because there are large unobserved differences. Basing an analysis on trends mitigates this risk because for it to affect our results requires that the unobserved differences in case mix between the two systems are changing over time.

This then suggests that there are two avenues to explore further. The first is to determine whether the alternatives to care in hospital setting have diverged in the two countries. If for example alternative settings to which terminally ill patients can be discharged have expanded faster in England than in Scotland we would observe the kind of differential trend of in-hospital mortality established by our analysis. The second, more worrying possibility is that there remains some element of the difference in trend that relates to the efficacy of hospital treatments in the two countries. The details of such potential *quality of care* differences are for clinicians and practitioners who are familiar with hospital treatments of specific conditions to explore, considering any changes in practice or performance targets relevant to them, e.g. during the period of analysis Scotland had targets regarding access and treatment of specific patient groups (21).

For peer review only

REFERENCES

1. Coombes R. Experts disagree about usefulness of hospital mortality data. BMJ : British Medical Journal. 2014;349.

2. Holland W, McKee M. Can hospital mortality comparisons ever be fit for purpose? BMJ : British Medical Journal. 2013;347.

3. Lilford R, Pronovost P. Using hospital mortality rates to judge hospital performance: a bad idea that just won't go away. BMJ. 2010;340.

4. Hogan H, Zipfel R, Neuburger J, Hutchings A, Darzi A, Black N. Avoidability of hospital deaths and association with hospital-wide mortality ratios: retrospective case record review and regression analysis. BMJ : British Medical Journal. 2015;351.

5. Black N. Assessing the quality of hospitals. BMJ. 2010;340.

6. van Gestel YR, Lemmens VE, Lingsma HF, de Hingh IH, Rutten HJ, Coebergh JW. The Hospital Standardized Mortality Ratio Fallacy: A Narrative Review. Medical Care. 2012;50(8):662–7.

7. Girling AJ, Hofer TP, Wu J, Chilton PJ, Nicholl JP, Mohammed MA, et al. Case-mix adjusted hospital mortality is a poor proxy for preventable mortality: a modelling study. BMJ Quality & Safety. 2012;21:1052-6.

8. Stewart K, Choudry MI, Buckingham R. Learning from hospital mortality. Clinical Medicine. 2016;16(6):530-4.

9. Hawkes N. Patient coding and the ratings game. BMJ. 2010;340.

10. Bottle A, Jarman B, Aylin P. Hospital Standardized Mortality Ratios: Sensitivity Analyses on the Impact of Coding. Health Services Research. 2011;46(6pt1):1741-61.

11. Farrar S, Yi D, Sutton M, Chalkley M, Sussex J, Scott A. Has payment by results affected the way that English hospitals provide care? Difference-in-differences analysis. BMJ. 2009;339.

12. Bradford E. Death is 'core business' of Scottish hospitals, study finds. BBC News19 March 2014.

13. Office for National Statistics. Vital Statistics: Population and Health Reference Tables.

14. Office for National Statistics. Life Expectancy at Birth and at Age 65 by Local Areas in England and Wales.

15. Office for National Statistics. Life Expectancy at Birth and at Age 65 for the UK and Local Areas in Scotland.

16. Hawe E, Cockcroft L. OHE Guide to UK Health and Health Care Statistics. 2013.

17. Wanhainen A, Hultgren R, Linné A, Holst J, Gottsäter A, Langenskiöld M, et al. Outcome of the Swedish Nationwide Abdominal Aortic Aneurysm Screening Program. Circulation. 2016;134(16):1141-8.

For peer review only

18. Health and Social Care Information Centre. Hospital Episode Statistics, Admitted Patient Care, England - 2013-14. 2015.

19. Information Services Division. Acute Hospital Activity and NHS Beds Information, Quarter ending March 2014 2014.

20. NHS Digital. Introduction to Healthcare Resource Groups.

21. NHSScotland Performance and Business Management. HEAT Targets due for delivery 2006-2015. 2014.

For peer review only

FIGURES

Figure 1. In Hospital Mortality Rate. Elective (LHS) and Emergency (RHS)
Note: the y-axis scales are different; LHS is 1/10 of RHS.

Figure 2. Crude Mortality Rate (LHS) and Life Expectancy (RHS)

Figure 3. In Hospital Mortality Rate. England/Scotland with 1997/98 = 100.

For peer review only

For peer review only

For peer review only

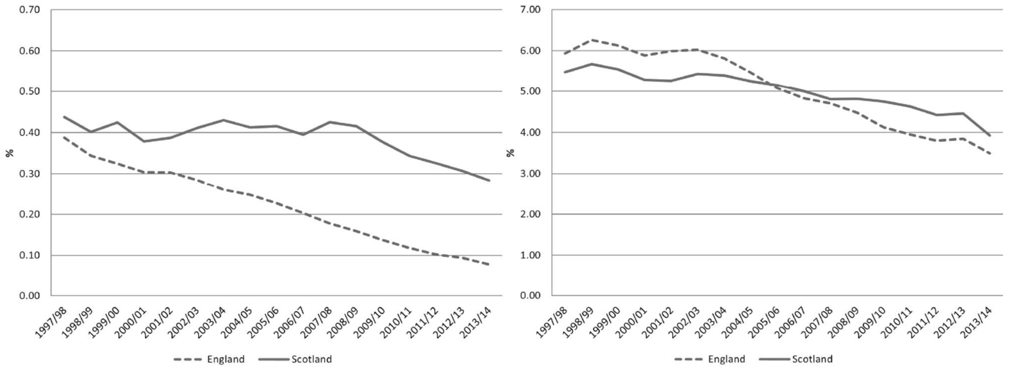

Figure 1. In Hospital Mortality Rate. Elective (LHS) and Emergency (RHS)
Note: the y-axis scales are different; LHS is 1/10 of RHS.

170x66mm (300 x 300 DPI)

For peer review only

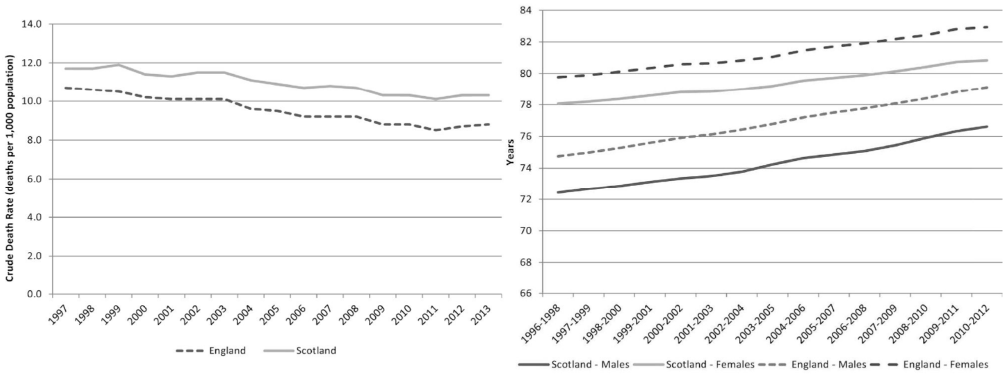

Figure 2. Crude Mortality Rate (LHS) and Life Expectancy (RHS)

170x66mm (300 x 300 DPI)

For peer review only

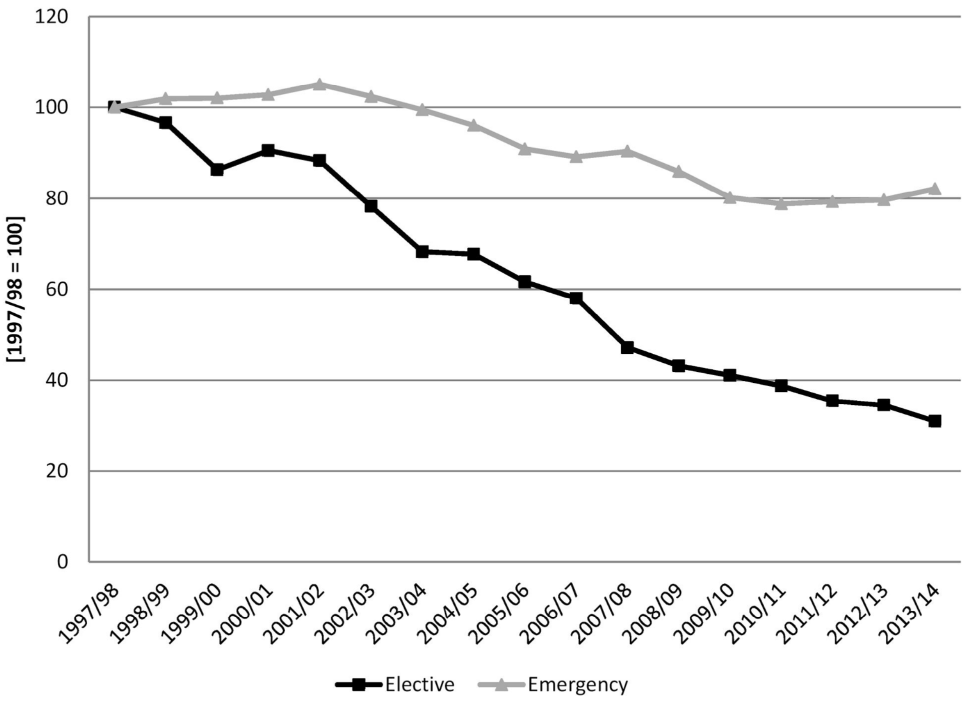

Figure 3. In Hospital Mortality Rate. England/Scotland with 1997/98 = 100

170x126mm (300 x 300 DPI)

For peer review only

BMJ Open

How do time trends in in-hospital mortality compare? A retrospective study of England and Scotland over 17 years using administrative data.

Journal:	*BMJ Open*
Manuscript ID	bmjopen-2017-017195.R4
Article Type:	Research
Date Submitted by the Author:	11-Oct-2017
Complete List of Authors:	Aragon Aragon, Maria Jose; University of York, Centre for Health Economics<br>Chalkley, Martin; University of York, Centre for Health Economics
**Primary Subject Heading**:	Health economics
Secondary Subject Heading:	Health services research
Keywords:	HEALTH ECONOMICS, Health economics < HEALTH SERVICES ADMINISTRATION & MANAGEMENT, Quality in health care < HEALTH SERVICES ADMINISTRATION & MANAGEMENT

SCHOLARONE™
Manuscripts

For peer review only

TITLE PAGE

Title: How do time trends in in-hospital mortality compare? A retrospective study of England and Scotland over 17 years using administrative data.

Authors:

María José Aragón (MJ Aragón) – Corresponding Author
mjma504@york.ac.uk
Research Fellow
Centre for Health Economics, University of York

Martin Chalkley (M Chalkley)
martin.chalkley@york.ac.uk
Professor
Centre for Health Economics, University of York

Centre for Health Economics
Alcuin 'A' Block
University of York
Heslington, York
YO10 5DD UK

Authors' Contributions:
MJ Aragón and M Chalkley defined the research question and type of analysis required, MJ Aragón performed the statistical analysis and MJ Aragón and M Chalkley analysed the results and wrote the article.

Acknowledgements
The Hospital Episode Statistics are copyright © 1997/98 - 2013/14, re-used with the permission of The Health & Social Care Information Centre. All rights reserved.
The Scottish Morbidity Record data was used with the permission of the Information and Statistics Division Scotland (ISD).

For peer review only

Declaration of competing interests

All authors have completed the Unified Competing Interest form at www.icmje.org/coi_disclosure.pdf (available on request from the corresponding author) and Dr. Chalkley and Dr. Aragon have nothing to disclose.

Data Sharing Statement

No additional data available.

Ethics Approval

No ethics approval was required for this study.

Funding Statement

This article was developed further to the NIHR funded project HS&DR - 11/1022/19.

For peer review only

ABSTRACT

Objectives

To examine the trends in in-hospital mortality for England and Scotland over a 17-year period to determine whether and if so to what extent the time trends differ after controlling for differences in the patients treated.

Design

Analysis of retrospective administrative hospital data using descriptive aggregate statistics of trends in in-hospital mortality and estimates of a logistic regression model of individual patient-level in-hospital mortality accounting for patient characteristics, case-mix and country and year specific intercepts.

Setting

Secondary care across all hospitals in England and Scotland from 1997 to 2013.

Population

Over 190 million inpatient admissions, either electively or emergency, in England or Scotland from 1997 to 2013

Data

Hospital Episode Statistics (HES) for England and the Scottish Morbidity Record 01 (SMR) for Scotland.

Main outcome measures

Separately for two admission pathways (elective and emergency) we examine aggregate time trends of the proportion of patients who die in hospital and a binary variable indicating whether an individual patient died in hospital or survived and how that indicator is influenced by the patient's characteristics, the year and the country (England or Scotland) in which they were admitted.

Results

In-hospital mortality has declined in both countries over the period studied, for both elective and emergency admissions but has declined more in England than Scotland. The difference in trend reduction is greater for elective admissions. These differences persist after controlling for patient characteristics and case-mix.

Conclusions

Comparing data at country level suggests questions about the roles performed by or functioning of their health care systems. We found substantial differences between Scotland and England in regard to the trend reductions in in-hospital mortality. Hospital resources are therefore being deployed increasingly differently over time in these two countries for reasons that have yet to be explained.

For peer review only

STRENGTHS AND LIMITATIONS OF THIS STUDY

- The first study to use comprehensive and extensive data on hospital admissions and discharges over a long period of time to study differences in in hospital mortality.
- Establishes a different perspective on in-hospital mortality – that of variation across health care systems over time and establishes that two neighbouring countries with otherwise similar health care systems have different time paths of in-hospital mortality.
- Uses detailed administrative records to control for variation in case-mix and patient characteristics.
- It is not possible to establish the potential causes of the different trends in in-hospital reported but potential causes are established as future avenues of research.

For peer review only

INTRODUCTION

In-hospital mortality has attracted a good deal of attention and concern when used as a proxy for hospital performance (1-6). The concern stems from an inability to disentangle consequences of treatment choices from the inherently different risks that patients' medical conditions pose (7-10). This debate however distracts from the potential knowledge that can be derived by studying in-hospital mortality at a more aggregate level (11). Hospital care is costly and a key resource in addressing a population's health care needs. It has been noted that death is a "core business" of hospitals (12) and hence understanding how that core business is changing – how much of the "business" of hospitals it accounts for – is a crucial aspect of health system planning and management.

Without some reference point it is impossible to determine whether an outcome such as declining in-hospital mortality is notable, or to be expected. Comparing two otherwise similar health care systems establishes each as a reference for the other. That reference is more powerful if analysis is conducted in trends. Differences in the levels of in-hospital mortality across different jurisdictions could easily be accounted for as the consequence of unobserved differences between their populations, health care needs and service organisation. However, these unobservable factors seem likely to follow common trends, so divergence in the trends of in-hospital mortality are more challenging to explain.

This, the first study of its kind, examines the trends in in-hospital mortality for England and Scotland over a 17-year period. We establish that "death as the core business of hospitals" has been declining faster in England than in Scotland over that period.

For peer review only

METHODS

Data

In both England and Scotland data are routinely collected on hospital inpatient activity through, respectively, Hospital Episode Statistics (HES) and the Scottish Morbidity Record (SMR). Both data sources report in terms of episodes (period under the care of one consultant), which are then converted into continuous inpatient spells (CIS) corresponding to the period of care that can include transfers within and between hospitals. We construct equivalent measures of CIS for both countries and distinguish between elective (including day cases) and emergency admissions, excluding maternity and regular attenders, using the type of admission of the first episode in the CIS. Both data sources report on the basis of financial years (1 April to 31 March) but for convenience we denote the financial year by its first calendar year. We examine over 190 million CIS from 1997 to 2013 using discharge information to determine whether the patient died in hospital or not.

Both data sources include the characteristics of a patient in regard to age, sex and the deprivations decile of their home address. We use these together with the Healthcare Resource Group (HRG) into which the patient's treatment fell to account for variation in case-mix.

Empirical Methods

The proportion of all CIS that end in death was calculated directly from the data sources, separately for each country, year and admission pathway.

After constructing a binary outcome variable (equal to 1 if the patient died in hospital and 0 otherwise) logistic regression analysis, separately for each admission pathway, was used to determine whether differences across jurisdictions persist after including covariates. To control for the potential influences of patient characteristics, case-mix and socio-economic circumstances the covariates included in the analysis were age (using five-year age bands indicators), sex (as an indicator equal to one for females), HRG indicators (there are more than 1000 different HRGs in the data) and deprivation decile indicators (1 being the most deprived and 10 the least deprived). Differences between countries were captured by country specific dummy variables and interactions between those and year dummy variables.

We ran logit regressions using Stata13.

For peer review only

RESULTS

Figure 1 shows the trend in the in-hospital mortality (CIS where patient died / Total CIS) for England and Scotland for elective and emergency admissions.

[FIGURE 1 HERE]

In Figure 1 it is apparent that in-hospital mortality has decreased in both countries, but has done so more quickly in England than in Scotland in both emergency and elective care. Over the same period the trends of overall mortality, measured by the crude mortality rate (deaths per 1,000 population), and life expectancy (in years) have been similar in both countries (13-15), see Figure 2. Whilst overall spending per head on hospital care is higher in Scotland it has followed a similar (increasing) trend as in England (16).

[FIGURE 2 HERE]

Next we describe the relative, England/Scotland, in-hospital mortality rate (CIS where patient died / Total CIS), again separately for Elective and Emergency admissions. Figure 3 shows the ratio of the in-hospital mortality rates for both countries, normalising to 100 the initial year, and clearly shows the relative change in the in-hospital mortality rates.

[FIGURE 3 HERE]

Next we determine whether these crude, unadjusted differences persist once we account for the different characteristics of the patients that are being treated in the two countries, specifically; age, sex, disease proxies and deprivation level.

Table 1 shows the descriptive statistics of the variables used for the regression analysis. Over the period of analysis in-hospital mortality has been higher for emergency than for elective admissions, that emergency admissions' patients are more likely to be males, are younger, and more likely to come from the highest deprivation decile than elective admissions.

Table 1. Descriptive Statistics

	Mean	Std. Dev.	Mean	Std. Dev.
Elective	**England**		**Scotland**	
**% Died in Hospital**	0.21	4.62	0.40	6.32
**% Males**	46.86	49.90	45.31	49.78
**Age**	54.43	21.26	53.73	21.03
**% Decile 1**	10.26	30.35	12.06	32.57
**% Decile 10**	9.00	28.62	8.21	27.46
**Number of Observations**	100,945,785		9,886,856	
Emergency	**England**		**Scotland**	
**% Died in Hospital**	4.98	21.75	5.01	21.82
**% Males**	48.03	50.00	49.14	49.99
**Age**	50.55	28.32	52.60	26.39
**% Decile 1**	14.70	35.41	15.85	36.52
**% Decile 10**	7.11	25.69	6.21	24.13
**Number of Observations**	74,048,633		8,259,572	

For peer review only

Table 2 shows regression results, presented as relative odd ratios between England and Scotland, this presentation was chosen to simplify the results table and focus on the question of interest: is the reduction in in-hospital mortality rates different between the countries after controlling for patient and CIS characteristics?

The results in Table 2 confirm what is observed in Figures 1 and 3, the reduction in in-hospital mortality in England has been faster than that of Scotland throughout the period, even after controlling for patient and CIS characteristics. The results are reported as odd-ratios, showing the relative difference between the two countries in each period, e.g. the first row says that in the initial year of the analysis Elective admissions were 11% lower in England than in Scotland and Emergency admissions were 3% higher. For *Electives* England starts with a lower in-hospital mortality rate (coefficient in first row is less than one and significant), then there is no clear trend in the difference between the countries until there is no significant difference between the two countries (non-significant coefficient in 2001/02) and then the difference with Scotland increases over time (coefficient becomes smaller over time) until in the last year in-hospital mortality for Elective admissions in England is around one third of that in Scotland. For *Emergencies* England started with a higher in-hospital mortality rate (coefficient in top row is greater than one and significant) and the difference between the two countries first increased (coefficients become greater) and then decreased until there is no difference between them (non-significant coefficient in 2007/08) and then England's in-hospital mortality rate continues to reduce relative to that of Scotland (coefficients smaller than one and significant from 2008/09 onwards) until being around 27% lower in the last year.

As it can be expected for a study comparing two specific countries, these results are not readily generalisable; the comparison of two specific countries, with similar health care systems, will yield a set of results which may or may not correspond to those obtained by comparing any other pair of countries. However, we have established a method of comparison which can apply in any circumstances in which there are suitable data.

For peer review only

Table 2. Logit Regression Results. Dependent Variable: Prob(Death). Relative OR England vs. Scotland

	Elective		Emergency	
**1997/98**	0.890	***	1.027	***
	(0.852-0.930)		(1.012-1.043)	
**1998/99**	0.929	***	1.096	***
	(0.889-0.971)		(1.079-1.113)	
**1999/00**	0.898	***	1.105	***
	(0.859-0.938)		(1.088-1.122)	
**2000/01**	0.952	**	1.105	***
	(0.909-0.996)		(1.088-1.122)	
**2001/02**	0.982		1.121	***
	(0.938-1.029)		(1.104-1.138)	
**2002/03**	0.941	**	1.095	***
	(0.899-0.986)		(1.078-1.111)	
**2003/04**	0.834	***	1.094	***
	(0.797-0.873)		(1.078-1.111)	
**2004/05**	0.776	***	1.069	***
	(0.741-0.813)		(1.053-1.085)	
**2005/06**	0.727	***	1.029	***
	(0.695-0.761)		(1.014-1.045)	
**2006/07**	0.675	***	1.044	***
	(0.644-0.707)		(1.028-1.060)	
**2007/08**	0.560	***	1.002	
	(0.535-0.586)		(0.987-1.017)	
**2008/09**	0.534	***	0.933	***
	(0.511-0.559)		(0.919-0.947)	
**2009/10**	0.511	***	0.868	***
	(0.487-0.536)		(0.855-0.881)	
**2010/11**	0.460	***	0.845	***
	(0.438-0.484)		(0.832-0.858)	
**2011/12**	0.403	***	0.854	***
	(0.383-0.424)		(0.841-0.867)	
**2012/13**	0.370	***	0.827	***
	(0.351-0.390)		(0.815-0.840)	
**2013/14**	0.329	***	0.831	***
	(0.312-0.348)		(0.818-0.844)	
**Dummy Variables Included as Controls:**				
**Age Group**	YES		YES	
**Gender**	YES		YES	
**Deprivation Decile**	YES		YES	
**HRG**	YES		YES	
**Number of Observations**	110,832,641		82,308,205	

Notes: 95% CIs in parenthesis. *** and ** indicate 1% and 5% significance, respectively. All regressions include a constant.

For peer review only

DISCUSSION

This study shows that in-hospital mortality for both elective and emergency admissions has been on a 17-year declining trend in both England and Scotland but that trend reduction has been greater in England. This remains the case after controlling for case mix and population characteristics.

We have used comprehensive and extensive data on hospital admissions and discharges over a long period of time, providing details of more than 190 million admissions. These data have been adjusted so as to be able to compare two similar health care systems so that each can act as a benchmark for the other. Whilst we can establish the differences between the experiences of these two systems with our data, we have not established causal mechanisms for these differences.

Numerous previous studies have examined the variation of in-hospital mortality across different hospitals, focusing on the details and limitations of risk-adjustment. This study provides a different perspective – that of variation across health care systems. Whilst we cannot hope to replicate the detail or depth of previous studies that focus on particular treatments we do provide a much broader and comprehensive view.

That view suggests a number of important and unanswered questions that have great potential importance for policymakers. Why has the divergence in trend reduction in in-hospital mortality developed? In what ways are these two health care systems developing different roles for their hospitals? Should there be a concern in Scotland that in-hospital mortality is decreasing less slowly and is not substantially higher than in its near neighbour England?

Answering the first of these questions will involve a search for clinical factors that may have exerted a differential impact on in-hospital mortality trends in the two countries. There are a number of candidates for such clinical confounders including, for example, the differential timing of the introduction of screening programs for high mortality conditions such as abdominal aortic aneurysm (17, 18), and the associated use of endovascular repair. It is worth noting, however, that any one factor is likely to account for only a small fraction of the difference in aggregate trends.

The subsequent questions concern the impact of health system reform and policies once all clinical factors are accounted for. These are also for future research – but we can give some insight and some clues as to the possible answers. One key difference in the development of hospital-based health care in Scotland and England over the period studied has been the reform of financing undertaken in England. This has been shown to have resulted in an expansion of activity on a per-capita basis. That suggests that part of the explanation for what we have observed is that hospitals in England are treating more "less-sick" patients which would result in a lower propensity for patients to die in hospital simply by increasing the denominator. However, that seems unlikely to be the whole explanation because we have established that the reductions in mortality exist for *both* elective and emergency admissions and whereas the former would appear susceptible to "denominator" effect it is less easy to account for emergency admissions in this way. The reduction on in-hospital mortality could also be related to the reduction in the duration of hospital admissions (usually called 'length of stay', LoS) which both countries report in the period of analysis (19, 20). We use HRGs to adjust for case-mix, however HRGs are meant not only to group together patients with similar diagnosis/treatment but also with similar resource intensity (21). Since we adjusted for the kinds of treatments that are carried out, and the resources needed to deliver them, in England and Scotland it is also difficult to account for the differences in trends in terms of changing case-mix

For peer review only

unless our adjustment is substantially flawed because there are large unobserved differences. Basing an analysis on trends mitigates this risk because for it to affect our results requires that the unobserved differences in case mix between the two systems are changing over time.

This then suggests that there are two avenues to explore further. The first is to determine whether the alternatives to care in hospital setting have diverged in the two countries. If for example alternative settings to which terminally ill patients can be discharged have expanded faster in England than in Scotland we would observe the kind of differential trend of in-hospital mortality established by our analysis. The second, more worrying possibility is that there remains some element of the difference in trend that relates to the efficacy of hospital treatments in the two countries. The details of such potential *quality of care* differences are for clinicians and practitioners who are familiar with hospital treatments of specific conditions to explore, considering any changes in practice or performance targets relevant to them, e.g. during the period of analysis Scotland had targets regarding access and treatment of specific patient groups (22).

For peer review only

REFERENCES

1. Coombes R. Experts disagree about usefulness of hospital mortality data. BMJ : British Medical Journal. 2014;349.

2. Holland W, McKee M. Can hospital mortality comparisons ever be fit for purpose? BMJ : British Medical Journal. 2013;347.

3. Lilford R, Pronovost P. Using hospital mortality rates to judge hospital performance: a bad idea that just won't go away. BMJ. 2010;340.

4. Hogan H, Zipfel R, Neuburger J, Hutchings A, Darzi A, Black N. Avoidability of hospital deaths and association with hospital-wide mortality ratios: retrospective case record review and regression analysis. BMJ : British Medical Journal. 2015;351.

5. Black N. Assessing the quality of hospitals. BMJ. 2010;340.

6. van Gestel YR, Lemmens VE, Lingsma HF, de Hingh IH, Rutten HJ, Coebergh JW. The Hospital Standardized Mortality Ratio Fallacy: A Narrative Review. Medical Care. 2012;50(8):662–7.

7. Girling AJ, Hofer TP, Wu J, Chilton PJ, Nicholl JP, Mohammed MA, et al. Case-mix adjusted hospital mortality is a poor proxy for preventable mortality: a modelling study. BMJ Quality & Safety. 2012;21:1052-6.

8. Stewart K, Choudry MI, Buckingham R. Learning from hospital mortality. Clinical Medicine. 2016;16(6):530-4.

9. Hawkes N. Patient coding and the ratings game. BMJ. 2010;340.

10. Bottle A, Jarman B, Aylin P. Hospital Standardized Mortality Ratios: Sensitivity Analyses on the Impact of Coding. Health Services Research. 2011;46(6pt1):1741-61.

11. Farrar S, Yi D, Sutton M, Chalkley M, Sussex J, Scott A. Has payment by results affected the way that English hospitals provide care? Difference-in-differences analysis. BMJ. 2009;339.

12. Bradford E. Death is 'core business' of Scottish hospitals, study finds. BBC News19 March 2014.

13. Office for National Statistics. Vital Statistics: Population and Health Reference Tables.

14. Office for National Statistics. Life Expectancy at Birth and at Age 65 by Local Areas in England and Wales.

15. Office for National Statistics. Life Expectancy at Birth and at Age 65 for the UK and Local Areas in Scotland.

16. Hawe E, Cockcroft L. OHE Guide to UK Health and Health Care Statistics. 2013.

17. Jacomelli J, Summers L, Stevenson A, Lees T, Earnshaw JJ. Impact of the first 5 years of a national abdominal aortic aneurysm screening programme. British Journal of Surgery. 2016;103(9):1125-31.

For peer review only

18. ISD Scotland. AAA Screening [Available from: http://www.isdscotland.org/Health-Topics/Public-Health/AAA-Screening/.

19. Health and Social Care Information Centre. Hospital Episode Statistics, Admitted Patient Care, England - 2013-14. 2015.

20. Information Services Division. Acute Hospital Activity and NHS Beds Information, Quarter ending March 2014 2014.

21. NHS Digital. Introduction to Healthcare Resource Groups.

22. NHSScotland Performance and Business Management. HEAT Targets due for delivery 2006-2015. 2014.

For peer review only

FIGURES

Figure 1. In Hospital Mortality Rate. Elective (LHS) and Emergency (RHS)
Note: the y-axis scales are different; LHS is 1/10 of RHS.

Figure 2. Crude Mortality Rate (LHS) and Life Expectancy (RHS)

Figure 3. In Hospital Mortality Rate. England/Scotland with 1997/98 = 100.

For peer review only

For peer review only

For peer review only

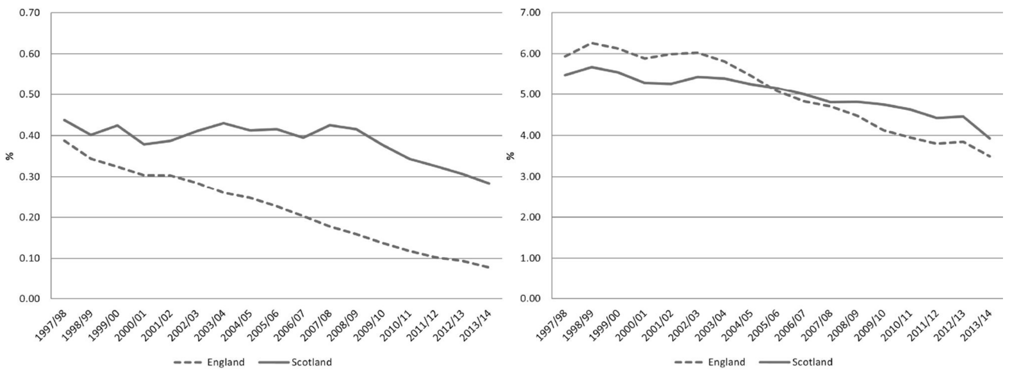

Figure 1. In Hospital Mortality Rate. Elective (LHS) and Emergency (RHS) Note: the y-axis scales are different; LHS is 1/10 of RHS.

170x66mm (300 x 300 DPI)

For peer review only

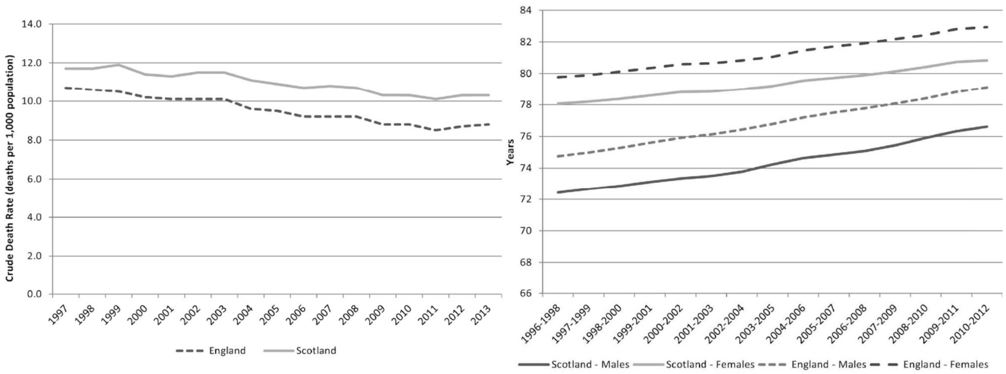

Figure 2. Crude Mortality Rate (LHS) and Life Expectancy (RHS)

170x66mm (300 x 300 DPI)

For peer review only

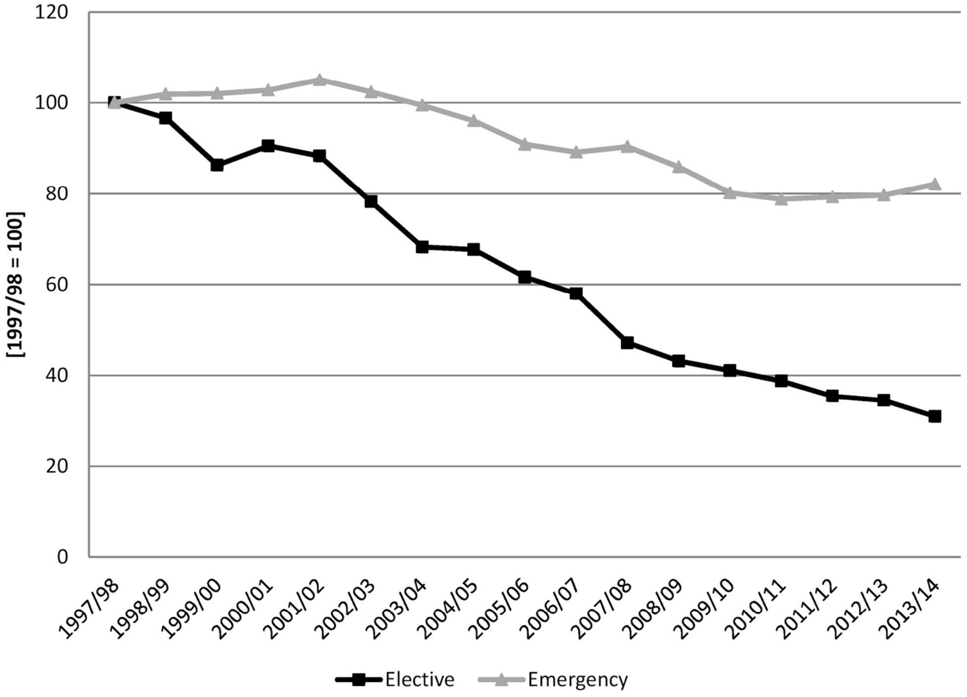

Figure 3. In Hospital Mortality Rate. England/Scotland with 1997/98 = 100

170x126mm (300 x 300 DPI)
